# Fertility loss: negative effects of environmental toxicants on oogenesis

**DOI:** 10.3389/fphys.2023.1219045

**Published:** 2023-08-04

**Authors:** Xiaoxi Yao, Weijing Liu, Yidong Xie, Mingrong Xi, Li Xiao

**Affiliations:** ^1^ Department of Obstetrics and Gynecology, West China Second University Hospital of Sichuan University, Chengdu, Sichuan, China; ^2^ Key Laboratory of Birth Defects and Related Diseases of Women and Children (Sichuan University), Ministry of Education, Chengdu, Sichuan, China; ^3^ Breast Center, West China Hospital, Sichuan University, Chengdu, Sichuan, China

**Keywords:** oogenesis, heavy metal, endocrine disruptors, smoke, agrochemicals

## Abstract

There has been a global decline in fertility rates, with ovulatory disorders emerging as the leading cause, contributing to a global lifetime infertility prevalence of 17.5%. Formation of the primordial follicle pool during early and further development of oocytes after puberty is crucial in determining female fertility and reproductive quality. However, the increasing exposure to environmental toxins (through occupational exposure and ubiquitous chemicals) in daily life is a growing concern; these toxins have been identified as significant risk factors for oogenesis in women. In light of this concern, this review aims to enhance our understanding of female reproductive system diseases and their implications. Specifically, we summarized and categorized the environmental toxins that can affect oogenesis. Here, we provide an overview of oogenesis, highlighting specific stages that may be susceptible to the influence of environmental toxins. Furthermore, we discuss the genetic and molecular mechanisms by which various environmental toxins, including metals, cigarette smoke, and agricultural and industrial toxins, affect female oogenesis. Raising awareness about the potential risks associated with toxin exposure is crucial. However, further research is needed to fully comprehend the mechanisms underlying these effects, including the identification of biomarkers to assess exposure levels and predict reproductive outcomes. By providing a comprehensive overview, this review aims to contribute to a better understanding of the impact of environmental toxins on female oogenesis and guide future research in this field.

## 1 Introduction

In recent years, a global decline in fertility rates and a high incidence of human infertility has been observed (GBD, 2019 Demographics Collaborators, 2020; Vollset et al., 2020). The World Health Organization (WHO, 2023) reported an estimated 17.5% lifetime prevalence of 2022 global infertility cases, and approximately one in six people experienced infertility at some stage in their lives; ovulatory disorders, the most common cause, account for approximately 25% of all infertility diagnoses (Carson and Kallen, 2021). Oogenesis is a crucial step in the development of female ovulatory function and is an important factor affecting fertility. Any abnormalities in either link can lead to defective oogenesis; for example, some environmental contaminants may interfere with the development of oocytes at the premeiotic or early prophase periods, resulting in chromosomal aberrations in the ova and affecting women’s reproductivity consequently (Foster and Hughes, 2011).

**FIGURE 1:**
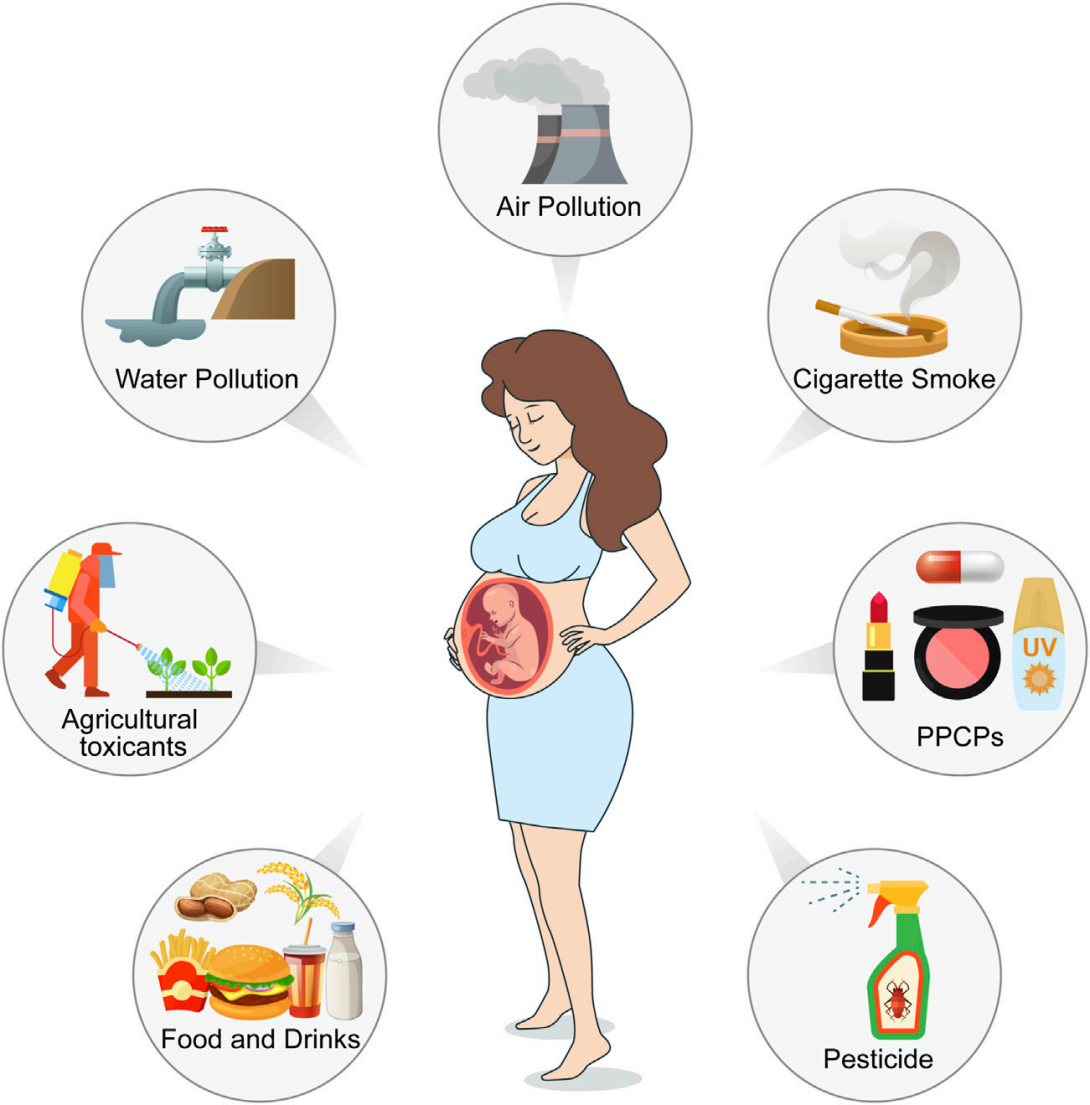
Pathways of environmental toxicants to females in reproductive age and female fetuses. Environmental pollution from air, water, and soil, as well as pesticides, reveals a reduction in oocyte survival, viability, and development. Exposure to cigarette smoke during pregnancy affects ovarian and liver function in female fetuses. Environmental toxicity presented in food and drinks, as well as in their packaging, blocks meiosis, interferes with spindle dynamics and increases aneuploidy. Pharmaceuticals and personal care products (PPCPs) inhibit oocyte meiosis, reduce oocyte quality and maturation, and promote apoptosis. (Created with Adobe Illustrator 26.0; Adobe).

Environmental toxicants are a class of chemical or biological substances capable of compromising human health, including heavy metals, cigarette smoke, pesticides, plastic products, pharmaceuticals and personal care products (PPCPs), food toxicants, and fluorides. Females are exposed to these toxicants through environmental pollution, food, cosmetics, and agricultural and industrial products (Figure 1). These ubiquitous toxicants may be implicated in ovarian and oocyte development via anatomical abnormalities and endocrinological dysfunction. For example, some endocrine disruptors, such as bisphenol A (BPA), have been reported to be crucial risk factors for endometriosis, endometrial cancer, polycystic ovary syndrome, and other estrogen-dependent diseases (Kandaraki et al., 2011; Priya et al., 2021; Stavridis et al., 2022; Stephens et al., 2022); furthermore, it may affect oocyte maturity, fertilization, and implantation, leading to higher infertility and miscarriage rates (Bloom et al., 2011; Ehrlich et al., 2012b; 2012a; Caserta et al., 2013). Heavy metal concentrations in the human body are associated with female reproductive disorders, preterm births, and stillbirths (Rzymski et al., 2015). It has been shown that heavy metals, such as cadmium, accumulate in embryos from the four-cell stage and inhibit progression to the blastocyst at a higher dosage, which may reduce the possibility of a successful pregnancy (Thompson and Bannigan, 2008).

**FIGURE 2:**
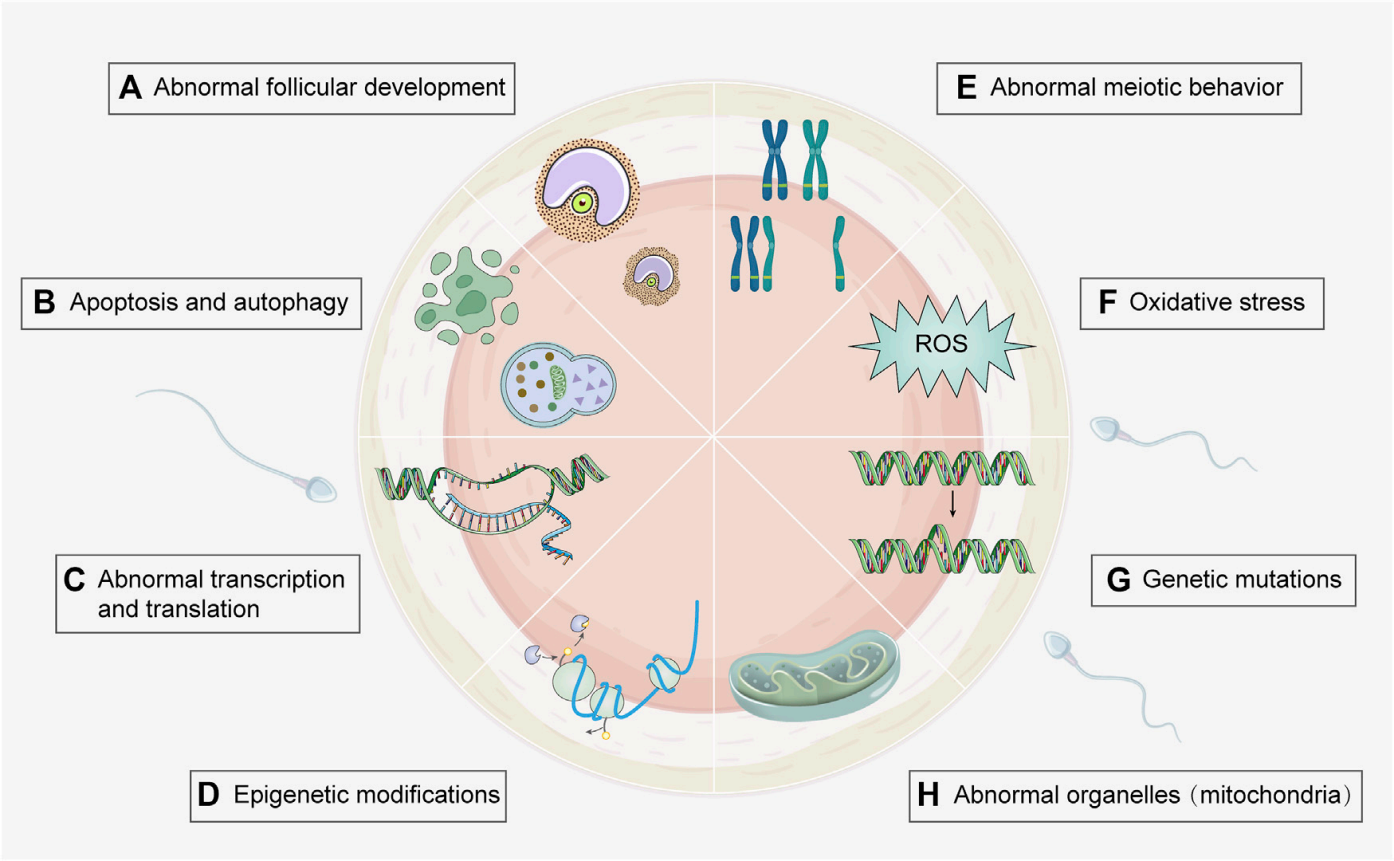
Toxic mechanisms of environmental toxicants on oogenesis process. Several genetic and molecular mechanisms involved in environmental toxicity can be underlying their effects on oogenesis. Associated mechanisms include **(A)** Abnormal follicular development (e.g., heavy metal, cigarette smoke, and PPCPs); **(B)** Apoptosis and autophagy (e.g., heavy metal, cigarette smoke, agricultural toxicants, food toxicant, and PPCPs); **(C)** Abnormal transcription and translation (e.g., agricultural toxicants); **(D)** Epigenetic modification (e.g., agricultural and food toxicants); **(E)** Abnormal meiotic behavior (e.g., heavy metal, cigarette smoke, agricultural and food toxicants); **(F)** Oxidative stress (e.g., heavy metal, cigarette smoke, food toxicants, and fluorides); **(G)** Genetic mutations (e.g., cigarette smoke and agricultural toxicants); **(H)** Abnormal organelles (e.g., agricultural and industrial toxicants). Detailed toxicants and associated mechanisms are provided in Supplementary Table S1. (Created with Adobe Illustrator 26.0; Adobe).

Pregnant women in the United States are reported to be exposed to 43 or more chemical toxins (Woodruff et al., 2011), which can elicit detrimental effects on fetal development and maternal health. Therefore, it is vital to investigate the effects of environmental toxicants on oogenesis. Because conditions such as polycystic ovary syndrome (PCOS) and premature ovarian insufficiency involve multiple risk factors, it is difficult to establish the role of toxicants. This review mainly focuses on the effects of environmental toxicants on female oogenesis, including the types of toxicants and the mechanisms that cause damage (Figure 2), in order to gain a better understanding and provide guidance for future research.

## 2 The process and influencing factors of oogenesis

### 2.1 The multiplication, growth, and maturation of the oocyte

**FIGURE 3:**
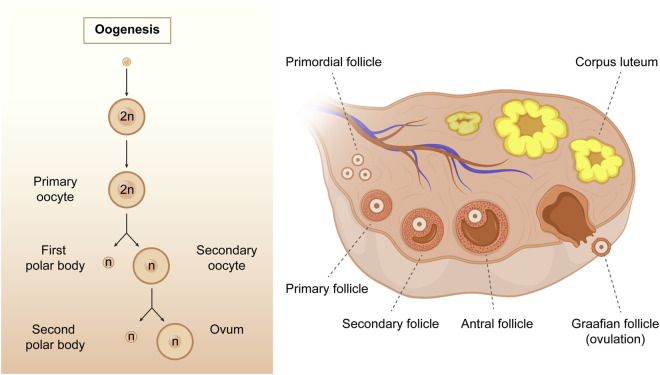
The process of oogenesis. Oogenesis starts with a transformation of the oogonia into the primary oocytes. During the meiotic process, only one mature oocyte will be produced, along with polar bodies through meiosis. As follicular development progresses, the primary follicles gradually develop into secondary follicles, antral follicles, and Graafian follicles. (Created with Adobe Illustrator 26.0; Adobe).

Oogenesis is a complex process by which female germ cells undergo a series of developmental changes to become mature oocytes. During the multiplication phase, primordial germ cells undergo mitotic division to form a large pool of oogonia that serve as precursors for primary oocytes (Gosden, 2002; Bukovsky et al., 2005). Oogonia multiplies mitotically and differentiates into primary oocytes, which are formed when oogonia undergo the first stage of meiosis. The growth phase is characterized by a significant increase in oocyte size, follicle formation, and accumulation of cytoplasmic components that support the growth and development of the embryo (Schultz et al., 1979). The hormonal regulation of oogenesis is critical, particularly during the growth phase of primary oocytes; the hypothalamus-pituitary-gonadal axis is activated during puberty, resulting in increased estrogen and follicle-stimulating hormone production (Spaziani et al., 2021). During the meiotic phase, primary oocytes undergo meiosis I and II. Meiosis I results in the formation of two haploid cells: a smaller first polar body (PB1) and a larger secondary oocyte. Meiosis II is arrested during metaphase II (MII) until fertilization. Ultimately, the mature ovum and polar bodies were formed (Figure 3). The mature ovum is released during ovulation and can be fertilized by sperm to form a zygote.

### 2.2 Factors affecting the process

Metabolism plays a pivotal role in the absorption of chemicals, and alterations in metabolism during pregnancy may render the developing fetus susceptible to various environmental toxins (Singh L. et al., 2020). The early stage of pregnancy is particularly delicate; it represents a critical window for fetal organogenesis. Exposure to various environmental toxins can disrupt the delicate balance between signaling pathways and gene expression that regulates oogenesis (Sadeu and Foster, 2011b; Sánchez and Smitz, 2012). Environmental toxins, like heavy metals, pesticides, and endocrine disruptors, have been proven to interfere with the proliferation and differentiation of primordial germ cells during the multiplication phase of oogenesis. Exposure to certain pesticides is linked to a decrease in the number of primordial germ cells and the size of the primordial follicle pool (Legoff et al., 2019), ultimately reducing the number of oocytes available for ovulation. Additionally, endocrine disruptors such as BPA, phthalates, and polychlorinated biphenyls (PCBs) alter the balance of hormonal signaling pathways that regulate oogenesis (Liu S.-Z. et al., 2016; Wang et al., 2016; Gonsioroski et al., 2022).

During the growth phase, environmental toxicants disrupt the hormonal balance in the reproductive system, altering the time and progression of oogenesis; particularly, polycyclic aromatic hydrocarbons (PAHs) exposure can interfere with the proper functioning of the hypothalamic-pituitary-gonadal axis, which regulates the production and release of sex hormones (Bolden et al., 2017; Yin et al., 2017). This disruption can lead to delayed onset of puberty, changes in menstrual cycles, and impaired fertility.

Several studies have reported that exposure to environmental toxicants during the meiotic phase can cause chromosomal aberrations, meiotic spindle defects, and abnormal distribution of chromosomes during cell division, resulting in aneuploidy, which is the presence of an abnormal number of chromosomes in a cell (Coticchio et al., 2015; Ding et al., 2020; Zhang et al., 2020). Aneuploidy is a significant cause of pregnancy loss and developmental disorders such as Down syndrome (MacLennan et al., 2015). Environmental toxicants have harmful effects on oogenesis and meiosis, leading to adverse reproductive outcomes; therefore, it is essential to identify and minimize exposure to environmental toxicants to protect reproductive health and ensure reproduction.

## 3 Environmental toxicants and oogenesis

### 3.1 Heavy metals

Oogenesis requires the involvement of various enzymes and trace elements; therefore, metals such as zinc (Zn), iron (Fe), and copper (Cu) are essential in oogenesis. Notably, there are two sides to these essential metal ions; their high levels have toxic effects on the oocytes (Cardoso–Jaime et al., 2022) and may be associated with female infertility (Li et al., 2020). Additionally, heavy metals, such as cadmium (Cd), lead (Pb), mercury (Hg), and Cu, can damage reproductive health as common environmental toxicants, and arsenic (As) is a heavy metal analog often included in studies. The mechanisms are summarized in Supplementary Table S1.

#### 3.1.1 Cadmium

Cd is a heavy metal endocrine disruptor. Numerous animal studies and epidemiological investigations have revealed that Cd abundance in the atmosphere, drinking water, food, and soil is hazardous to health and female fertility; it accumulates in the ovaries and affects ovarian function (Wang et al., 2015).

Research has revealed that Cd exposure reduces the number of oocytes, inhibits meiosis in oocytes, and reduces oocyte quality (Zhu et al., 2018). The underlying mechanisms include cytoskeletal organization, mitochondrial function, and histone modifications. Cd exposure inhibited meiosis by interfering with spindle assembly, chromosomal alignment, and actin cap development. Chronic Cd exposure has been found to disrupt chromosomal alignment and kinetochore-microtubule attachment, leading to the emergence of aneuploid oocytes, significantly reducing the ability of female mice to conceive (Dong et al., 2020).

Oocyte quality declines due to increased reactive oxygen species (ROS) levels and apoptosis induced by exposure to Cd. These changes resulted in abnormal mitochondrial distribution, a lack of energy, and DNA damage (Cheng et al., 2019). Additionally, Cd exposure affects the levels of global histone demethylation (H3K9me2), deacetylation (H4K12ac), lysine methylations (H3K9me3), acetylations (H3K9ac), and the global DNA methylation (5mC) in oocytes; these histone modifications are greatly associated with oocyte dysplasia and, consequently, the failure of embryonic development (Zhu et al., 2018; Cheng et al., 2019). Furthermore, it is reported that Cd interferes with the cyclical changes in maturation promoting factor (MPF) activity by altering the expression of MPF-related genes, which delays meiosis in oocytes (Liu et al., 2018).

#### 3.1.2 Arsenic

As and its compounds are extensively used in industry and agriculture (Chandravanshi et al., 2018). It is a toxic substance that affects the structure and function of female gonads, significantly reducing female fertility. The available results suggest that As inhibits oocyte meiosis and embryonic development. Numerous studies have demonstrated that arsenic trioxide significantly reduces mitochondrial DNA (mtDNA) copy number, causes mitochondrial damage, increases ROS levels, and decreases adenosine triphosphate (ATP) content (Zhang et al., 2011). Other studies have had similar results, and oocyte autophagy has been reported. These mechanisms can lead to a decrease in oocyte quality and quantity and cause embryonic arrest (Ommati et al., 2020; Kang et al., 2022). Additionally, aberrant expression of the global histone lysine trimethylation (H3K4me3), H3K9me3, and 5mC was observed in arsenic-exposed oocytes, indicating that As interferes with meiotic processes in oocytes by influencing DNA methylation and histone modifications. Studies have consistently reported that chronic As exposure in parental generations leads to cross-generational genotoxicity and global DNA methylation changes and is associated with reproductive defects in rats, implying that chronic As exposure may affect the health of offspring (Nava-Rivera et al., 2021).

#### 3.1.3 Lead

Pb is a metal that enters the human body through various routes, including water, air, and soil contamination. It poisons the blood and the nervous system and is associated with lower live birth rates in women having assisted reproductive treatments (Butts et al., 2021). Few studies have investigated the effects of Pb exposure on the female reproductive system. A comparison of Cd with Pb revealed a reduction in oocyte viability and development; however, Pb was significantly less toxic than Cd (Nandi et al., 2010; Slaby et al., 2017). Furthermore, Pb exposure caused histopathological and ultrastructural changes in oocytes and ovarian tissues. Simultaneously, the activities of catalase, glutathione peroxidase, total superoxide dismutase, and glutathione-S-transferase decreased, and malondialdehyde levels increased in the ovaries of mice. Additionally, Pb activates the Nrf2/Keap1 pathway, inhibiting oocyte maturation and fertilization by inducing oxidative stress (Jiang X. et al., 2021). Notably, when the Pb level was increased to 10 μg/mL, over 50% of the oocytes died (Nandi et al., 2010).

#### 3.1.4 Mercury

Hg is a common heavy metal used in industrial, household, and cosmetic products. Hg gas and its compounds are highly toxic and enter the body via respiratory inhalation, ingestion, and skin contact, causing systemic damage. Hg exposure can be assessed by measuring the levels of Hg in blood, urine, and hair; its exposure is strongly associated with biochemical pregnancy and live birth rates in women undergoing *in vitro* fertilization (IVF) (Butts et al., 2021). Hg concentrations in hair may be negatively associated with human oocyte production; however, some studies have concluded that hair Hg levels are not associated with ovarian stimulation outcomes, IVF pregnancy, or live birth rates (Dickerson et al., 2011; Wright et al., 2015). Investigations in a zebrafish model demonstrated that zebrafish ovaries in the Hg-exposed group revealed a reduction in the number of previtellogenic and early vitellogenic oocytes and atresia of the previtellogenic oocytes; however, there was no effect on the size of mature oocytes, suggesting that the toxic effects of Hg on oocytes are in the early stages of adulthood (Patel et al., 2022). The histomorphology of the ovary was altered by Hg vapor inhalation, resulting in a reduction in the number of primordial, primary, and graafian follicles and a significant increase in the total volume of atretic follicles. Additionally, irregularities in follicle and oocyte boundaries and edematous degeneration of follicular granulosa cells have been observed in rats after Hg vapor intervention (Altunkaynak et al., 2016). Existing studies have demonstrated the effects of Hg exposure on oogenesis; however, the exact mechanism of its toxic effects is unclear. Further research is needed to assess the impact of Hg exposure levels on fertility outcomes in women during their fertile period.

#### 3.1.5 Copper

Cu is an essential trace element in the human body. The Cu transport system is essential for follicular development in pigs, and copper deficiency negatively affects follicular development. In an *in vitro* experiment, administering appropriate concentrations of Cu reduced oxidative stress and apoptosis, promoted oocyte maturation, and improved oogenesis and blastocyst formation rates (Choi et al., 2021; 2022). However, a study revealed that increased Cu levels in the follicular fluid might induce abnormal steroid synthesis and affect follicular development in patients with PCOS (Sun et al., 2019). The toxicity of Cu in the female reproductive system is gradually being considered; high Cu levels damage oocyte chromosome structure and cause abnormal dynamics of actin distribution, activating the Nrf2 signaling pathway and leading to increased ROS levels; ultimately, this leads to reduced oocyte quality in pigs (Chen et al., 2021; Zhan et al., 2022). Similar results were obtained in experiments using copper sulfate to treat sheep oocytes; glutathione supplementation corrected copper-induced oocyte damage (Ren et al., 2023). Excess Cu has been shown to have a toxic effect on oogenesis in animal studies. However, there is a lack of evidence in humans.

#### 3.1.6 Other metal

In addition to the common heavy metal toxins, there are a growing number of studies on the toxicity of other metals. Thallium may affect oogenesis and is associated with early embryonic abortion (Liang et al., 2022). Recently, metallic tin compounds have received attention as important environmental contaminants. Tributyltin oxide (TBTO) exposure impairs oocyte maturation by inducing intracellular ROS accumulation, causing mitochondrial dysfunction, and disrupting cellular iron homeostasis; consequently, this reduces oocyte quality and interferes with embryonic developmental capacity (Xiao et al., 2021). Hexavalent chromium (CrVI) promotes oocyte apoptosis and alters cell cycle regulatory genes and proteins. Chromium (Cr) exposure during gestation causes premature ovarian failure in offspring F1 rats (Stanley et al., 2015).

In addition to occupational exposure, heavy metal toxins exhibit numerous exposure pathways. The reduction in toxicity (due to exposure to heavy metals) in reproduction is gradually being investigated. For instance, blueberry extracts have been shown to reduce the adverse effects of Cd on the ovaries (Izaguirry et al., 2017). Additionally, quercetin inhibits oxidative stress in germ cells (Mao et al., 2018), and glutathione antagonizes As damage in goat oocytes by preserving or maintaining mitochondrial function, gene expression, and anti-oxidative products (Ren et al., 2022). These studies explored the antioxidant properties of these pharmaceuticals in terms of their ability to mitigate heavy metal toxicity. Therefore, more preventive measures that can alleviate heavy metal toxicity should be explored, especially in foods with antioxidant components without side effects on humans.

### 3.2 Cigarette smoke

The toxic effects of cigarette smoke and its components on the reproductive system are extensively recognized. Exposure to cigarettes during pregnancy can affect ovarian and liver functions in female fetuses (Talbot and Lin, 2011; Fowler et al., 2014). The harmful components include PAH, nicotine, benzo(a)pyrene (BaP), pyrazine, 2-ethylpyridine, and 3-ethylpyridine. Recently, in addition to cigarette smoke, smokeless tobacco (ST) has similar toxic components and effects on the reproductive system (Laldinsangi, 2022), as presented in Supplementary Table S1.

#### 3.2.1 Smoke

Some observational studies in humans have concluded that smoking does not significantly affect oocyte quantity or quality (Budani et al., 2017; Fréour et al., 2018; Ozbakir and Tulay, 2020). However, smoking delays egg cleavage and affects early embryonic development. Additionally, researchers have identified an association between smoking and lower fertilization and pregnancy rates in women undergoing IVF (Fréour et al., 2013; Budani et al., 2017). In animal studies, cigarette smoke extracts have been found to cause premature luteinization of mouse follicles, reduced follicle survival, a diminished diameter of zona pellucida-free oocytes, and PB1 abnormalities in mice (Sadeu and Foster, 2011a; Mai et al., 2014). Impaired sinus follicle growth in mice exposed to cigarette smoke persists after smoking cessation (Paixão et al., 2012). Furthermore, although cigarette smoke exposure did not significantly reduce the number of oocytes, a thicker zona pellucida, and shorter and wider spindle bodies were observed (Jennings et al., 2011). If the parental generation is exposed to cigarettes during pregnancy, in that case, the F1 generation female mice show elevated levels of oxidative stress in somatic oocytes and changes in mid-term II spindle bodies, which is a hazard that persists into sexual maturity (Camlin et al., 2016). Changes in microRNAs (miRNAs) involved in the regulation of cell proliferation and apoptosis were observed in MII oocytes from cigarette smoke-exposed mice. Additionally, cigarette smoke exposure activated *CYP2E1* and oxidative stress, impairing oocyte development (Sobinoff et al., 2013; Budani et al., 2019). In addition to first-hand smoking, second-hand and third-hand smoke (THS) can harm women’s reproductive health. THS is the residual tobacco smoke that remains on surfaces or in dust after smoking. The main component, 1-(N-methyl-N-nitrosamino)1-(3-pyridinyl)-4-butanal (NNA), affects mouse oocyte survival; it alters epigenetic modifications by increasing the risk of DNA damage and abnormal spindle morphology, decreasing 5mC and histone lysine methylation (H3K4me2) levels, and inducing apoptosis through mitochondrial dysfunction and ROS accumulation (Liu et al., 2019b; 2019a).

#### 3.2.2 Nicotine

Nicotine is the primary toxic component of cigarettes, and its metabolite is cotinine. In an experiment using human fetal ovaries, the addition of nicotine significantly reduced the number of oocytes in fetal ovaries, indicating that nicotine may directly induce oocyte apoptosis (Cheng et al., 2018). Nicotine exposure is associated with reduced expression of mouse oocyte-specific genes, such as *NOBOX*, *LHX8*, *FIGLA*, and *SOHLH2*. In addition, mice injected with nicotine showed increased levels of oxidative stress and autophagy markers in ovarian cells, including upregulation of AMPKα-1, increased protein light chain 3 (LC3)-II/LC3-I ratio, and downregulation of AKT and mTOR (Wang et al., 2018). The metabolic products of cotinine have been found to reduce oocyte quality (Cheng et al., 2022); luteinizing hormone and follicle-stimulating hormone antagonize this effect (Liu W.-X. et al., 2021). Additionally, it has been suggested that melatonin increases MnSOD expression, reduces nicotine-induced intracellular ROS production, and maintains normal H3K4 and H3K9 demethylation and trimethylation in zygotic ovaries. These mechanisms enable melatonin to counteract damage to oocytes caused by nicotine and its metabolites (Wang et al., 2018; Liu et al., 2020; Cheng et al., 2022).

#### 3.2.3 Polycyclic aromatic hydrocarbons

PAHs originate from the incomplete combustion of many substances, and the main route of human exposure is cigarette smoke. PAHs inhibit meiosis and promote apoptosis of primary chicken germ cells (Ge et al., 2012). During the immature stage, PAH-exposed mice exhibit a significant increase in the number of luminal follicles and inhibit oocyte maturation and follicular atresia. Transcriptome analysis suggested that this may be related to the upregulation of the PI3K/Akt signaling pathway and disruption of the calcium signaling pathway (Guo et al., 2022). BaP is a PAH that is established to be carcinogenic and toxic to the reproductive system. BaP affects oocyte meiosis in mice and impairs the quantity and quality of oocytes in offspring (Zhang et al., 2018). Several studies have shown that BaP reduces mitochondrial content and ATPs synthesis and increases the accumulation of reactive oxidants and genomic DNA 5mC levels in offspring oocytes (Sadeu and Foster, 2011b; Sui et al., 2020; Malott et al., 2022). In addition, BaP exposure impairs the meiotic process in mouse oocytes by disrupting normal spindle assembly, chromosome arrangement, and mitotic microtubule attachment, leading to the production of aneuploid eggs. It significantly reduces fertilization rates in mice by reducing the number of sperm bound to the egg zona pellucida and interfering with the gamete fusion process (Einaudi et al., 2014; Zhang et al., 2018). Pyrazine, 2-ethylpyridine, and 3-ethylpyridine are harmful cigarette components, which inhibit oocyte maturation and affect sperm fertilization in women (Wu and Liu, 2012).

### 3.3 Agricultural and industrial toxicants

#### 3.3.1 Herbicides

Glyphosate-based herbicides are common herbicides; however, whether they are carcinogenic to humans is controversial. Glyphosate exposure has been suggested to impair oocyte quality in mid-stage II mice by disrupting microtubule tissue centers and chromosomes, significant depletion of intracellular zinc bioavailability, and enhanced accumulation of reactive oxygen clusters (Spinaci et al., 2020; Yahfoufi et al., 2020; Zhang et al., 2021). Low doses of glyphosate adversely affect oocyte maturation and early embryonic oogenesis by increasing the levels of ROS, spindle defects, and chromosome misalignment (Cao et al., 2021). Its adjuvant is used to increase its herbicidal effect, and available studies indicate that the addition of an adjuvant enhances its toxicity and increases oocyte ROS levels (E et al., 2022). Fenoxaprop-ethyl is a post-emergence herbicide with an aryloxyphenoxypropionate moiety designed to prevent fatty acid synthesis by inhibiting acetyl coenzyme A carboxylase. Exposure to FE disrupts the oocyte cytoskeleton and induces ROS accumulation, affecting meiosis (He et al., 2019b). Atrazine is commonly used as a herbicide; exposure of embryos to Atrazine affects meiosis prophase I, reduces the number of primordial follicles, and increases the incidence of polyzygotic follicles in adult mice (Gely-Pernot et al., 2017). Furthermore, exposure to Atrazine significantly downregulates MLH1 in *drosophila*, with transcriptional and translational defects, reduced double-strand breaks, and association complex formation; this leads to reduced fertility, reduced numbers of mature oocytes, abnormal ovarian follicle distribution, increased apoptosis, and abnormal embryonic development after fertilization in *Drosophila* (Vimal et al., 2019).

Generally, the toxic effects of herbicides on oocytes are evident. Some studies have investigated the drugs that can be used to treat this condition. Current evidence suggests that melatonin has a mitigating effect on the ototoxicity of glyphosate-based herbicides and fenoxaprop-ethyl, and its possible mechanisms include the prevention of glyphosate-based herbicides-induced oocyte deterioration and activation of its downstream signaling events (pERK/ERK) by protecting G-protein estrogen receptor (GPER/GPR30) expression and regulating the levels of regulatory hormones (He et al., 2019a; Cao et al., 2021; Zhang et al., 2021).

#### 3.3.2 Pesticides

Pesticides are a class of endocrine disruptors extensively used in crop production. Common categories of pesticides include organophosphorus pesticides, neonicotinoids, formate insecticides, and pyrethroids.

The organophosphorus pesticide, malathion, significantly reduces oocyte survival and affects the regulation of genes for transcription, translation-related proteins, and mitochondrial function, affecting early oogenesis and oocyte viability (Bonilla et al., 2008; Flores et al., 2017). Methyl parathion (MP) is the most commonly used class of organophosphorus pesticide. MP exposure reduces the number of primordial follicles and increases DNA damage in granulosa cells. Additionally, it exerts a significant inhibitory effect on oocyte nuclear maturation and is associated with spindle malformation. Furthermore, MP-treated oocytes showed higher cytoplasmic abnormalities and depleted glutathione levels (Nair et al., 2014; Satar et al., 2015). Gai et al. discovered that exposure to the organophosphorus pesticide diazinon (DZN) inhibited oocyte meiosis and reduced egg fertilization (Gai et al., 2022). Chlorpyrifos (CPF) is one of the most extensively used organophosphorus pesticides. Studies have shown that CPF exposure significantly reduces the extrusion rate of the PB1 and impaired MII oocytes. Exposure to 10 mM CPF disrupts meiotic cycle progression, leads to abnormal spindle and mitochondrial dysfunction and induces oxidative stress and apoptosis in porcine oocytes (Jiang Y. et al., 2021). 3-methyl-4-nitrophenol (PNMC), a degradation product of organophosphorus pesticides, significantly inhibits the resumption of meiosis in mouse oocytes (Han et al., 2018). Neonicotinoids are extensively utilized in modern agriculture. Additionally, thiamethoxam changes the expression of several oocyte genes associated with inflammation, apoptosis, and endoplasmic reticulum stress; it affects DNA integrity, promotes oxidative stress and endoplasmic reticulum stress, and induces apoptosis (Liu Y. et al., 2021). Acetamiprid (ACE) and imidacloprid are commonly used neonicotinoids. The effects of ACE and imidacloprid on porcine oocyte maturation *in vitro* have been reported previously. Irregular chromosomes were observed in the ACE- or imidacloprid-exposed groups and nuclear maturation rates were significantly reduced (Ishikawa et al., 2015). Formate insecticides are commonly used. Previous studies have shown that methomyl inhibits polar body extrusion in mouse oocytes. A significant increase in superoxide anion radicals in oocytes and a significant decrease in mitochondrial membrane potential in mid-stage II oocytes were observed in the methomyl-treated group, resulting in reduced mouse oocyte mass. After prolonged exposure to methomyl, mid-stage I mouse oocytes exhibit morphologically abnormal spindle bodies (He D. et al., 2022). Carbofuran (CF) has been banned in several countries and regions but remains an extensively used carbamate insecticide. CF induces programmed cell death dose-dependently and may alter spindle morphology by interacting with microtubules (MTs) assembly or impairing pericentriolar protein orientation. The inhibition concentration 50 (IC50) for 2000, 1,000, and 250 mM CF was calculated for the 6, 24, and 48 h incubation periods, respectively (Cinar et al., 2015). Pyrethroids are gradually replacing other insecticides. Cypermethrin, deltamethrin, and cyhalothrin are gradually replacing other insecticides, such as organophosphates. It is less toxic than traditional insecticides; however, it has been found to have adverse effects on animal fertility and may affect pregnancy outcomes in assisted reproduction (Tchounwou, 2004; Singh D. et al., 2020). Cypermethrin, deltamethrin, and cyhalothrin had no significant effect on oocyte survival at the concentrations tested. However, there was a concentration-dependent decrease in oocyte maturation rates in the experimental animals; additionally, abnormal spindle morphology and DNA double-strand breaks occurred. Furthermore, oxidative stress and apoptosis have been observed in the oocytes of mice exposed to deltamethrin (Petr et al., 2013; Jia Z.-Z. et al., 2019).

Specific types of insecticides have been found to affect egg cell production. Fipronil (FPN) is an extensively used phenylpyrazole insecticide that kills pests by blocking γ-aminobutyric acid (GABA)-gated chloride channels. FPN increases ROS levels and DNA damage during porcine oocyte maturation and induces apoptosis and cell cycle arrest (Zhou et al., 2019). Methoxychlor (MXC) is a common cytotoxic and genotoxic organochlorine pesticide; MXC negatively affects oocyte meiotic maturation, primarily by cellular ROS metabolism impairment (Liu et al., 2016). Rotenone inhibited mitochondrial complex I. Rotenone treatment induced mitochondrial dysfunction and failure of mitochondrial biosynthesis and inhibited the maturation of porcine oocytes *in vitro* (Heo et al., 2022).

Pesticides include pesticidal agents used in agricultural production and varieties used in domestic mosquitoes and insect control, with more routes of exposure. Propolis has been suggested to alleviate the toxic effects of MXC, and its mechanism of action may be related to its antioxidant potential (El-Sharkawy et al., 2014). Notably, vitamin E has a protective effect in rats exposed to cypermethrin, increases glucose uptake in follicle cells and oocytes, and inhibits the biosynthesis of the pro-apoptotic protein caspase-3 (Molavi et al., 2016.). Although pyrethroids have been extensively used as fewer toxic insecticides, the available evidence does not completely exclude the potential harm of such insecticides to female reproductive function or the female fetus, and further research is still needed in this area.

#### 3.3.3 Fungicides

Fungicides are agricultural toxicants used in the cultivation of a wide range of crops. Dexamethasone zinc (MNZ) significantly reduced the number of oocytes, oocyte maturation rate, fertilization rate, fertility rate, fertility, and embryo development in F1 generation mouse pups (Esmaiel et al., 2019). As an endocrine disruptor, it has been suggested to induce progesterone secretion from granulosa cells, inhibiting the luteinizing hormone peak during ovulation (Dinisri et al., 2021). Captan alters the expression of multiple genes in oocytes; it triggers DNA damage, autophagy, and apoptosis, induces oxidative stress and mitochondrial dysfunction, disrupts mitochondrial structure and distribution, and depolarizes membrane potential (He Q.-K. et al., 2022). Azoxystrobin (AZO) is an extensively used fungicide that exerts fungicidal activity by inhibiting mitochondrial respiration. AZO exposure impairs oocyte maturation by increasing oxidative stress and mitochondrial dysfunction and reducing the integrity and spindle formation of microtubule organizing centers (MTOCs) and chromosome alignment (Gao et al., 2022).

#### 3.3.4 Plasticizers

Plasticizers are increasingly used in domestic production. Di(2-ethylhexyl) phthalate (DEHP) is an extensively used plasticizer. As an endocrine disruptor, DEHP affects steroid production, sinus follicle growth, and primordial follicle production (Zhang et al., 2013; Zhou and Flaws, 2016; Gonsioroski et al., 2022). Animal studies have shown that DEHP exposure significantly reduces the number of primordial follicles during puberty and adulthood, possibly by accelerating the kinetic rate of follicle recruitment, decreasing or delaying imprinted gene methylation in oocytes, and increasing mid-term II spindle abnormalities in mature oocytes *in vitro* (Zhang et al., 2013). Maternal exposure to a mixture of phthalates affected folliculogenesis and steroidogenesis in F1 and F2 generation female rats and altered the expression of miRNAs in the F1 generation ovaries of female rats (Gonsioroski et al., 2022). DEHP exposure alters ovarian miRNA expression in F1 generation rats and reduces the expression of H3K4me3, estrogen receptor (ER) Ⅶ, progesterone receptor (PR), and Notch2 signaling components (Mu et al., 2015; Gonsioroski et al., 2022; Li et al., 2023). Furthermore, it has been found that the combination of DEHP exposure and a high-fat diet has a synergistic effect, significantly increasing synaptic defects in meiosis and affecting folliculogenesis in the F1 generation (Mirihagalle et al., 2019).

Dibutyl benzodicarboxylate (DBP) is an extensively used plasticizer that induces apoptosis in oocytes by increasing oxidative stress-induced DNA damage. DBP exposure during gestation disrupts the progression of meiotic prophase I in mouse oocytes, particularly from even-to coarse-lineage phases (Tu et al., 2019). In addition, DBP significantly reduced germinal vesicle breakdown and polar body extrusion in mouse oocytes and disrupted the cytoskeleton, and cortical granule-free domains of oocytes were disrupted (Li et al., 2019).

BPA is extensively used in the plastics industry, such as water bottles, containers, packaging, and toys, and is a common endocrine disruptor that causes delayed maturation of animal oocytes and significantly affects embryonic developmental potential (Ferris et al., 2016; Nakano et al., 2016; Saleh et al., 2021). Studies have shown that BPA affects the expression of AMH and AMH receptor II during oocyte maturation (Saleh et al., 2021). BPA activates the Gper/Egfr/Mapk3/1 pathway, which disrupts meiosis in zebrafish oocytes (Fitzgerald et al., 2015); it exerts toxic effects on oocytes by disrupting the cytoskeletal dynamics, inhibiting spindle stability, affecting epigenetic modifications, and inducing apoptosis and autophagy (Wang et al., 2016; Yang et al., 2020). In addition, BPA affects organelles in oocytes, causing mitochondrial dysfunction, abnormal mitochondrial distribution, abnormal endoplasmic reticulum distribution, abnormal Golgi apparatus structure, and lysosomal damage (Pan et al., 2021). Fluorene-9-bisphenol (BHPF), bisphenol S (BPS), and bisphenol AF (BPAF) are alternatives to BPA for the production of “BPA-free” plastics; however, they are harmful to living organisms. Studies have shown that BHFP inhibits the maturation of mouse oocytes *in vitro*, inhibits oocyte expansion at certain concentrations, and significantly reduces polar body excretion. Furthermore, they cause abnormal spindle assembly, reduced ATP levels, ROS accumulation, early apoptosis, and disturbed distribution of CGs in porcine oocytes (Ding et al., 2017; Jia Z. et al., 2019; Jiao et al., 2020). Similar to BPA, BPS can affect the expression of AMH and AMH receptors (Saleh et al., 2021). Notably, it may be not dose-dependent of the toxic effects of BPS, which affects the developmental quality of oocytes at a lower order of magnitude concentrations (Prokešová et al., 2020).

### 3.4 Pharmaceuticals and personal care products

#### 3.4.1 Nanoparticle

Nanoparticles (NPs) are materials with at least one dimension less than 100 nm and are extensively used in cosmetics, food, health, and personal care products (Santacruz–Márquez et al., 2021).

Zinc oxide nanoparticles (ZnO NPs) are among the most widely used nanomaterials in industrial and commercial products (Huang et al., 2022). ZnO NPs inhibit oocyte meiosis, reduce oocyte quantity and quality, and arrest early embryonic development by releasing zinc ions to produce cytotoxicity. ZnO NPs induce cytotoxicity by releasing zinc ions, which inhibit oocyte meiosis, reduce the number and quality of oocytes, and arrest early embryonic development. The mechanisms involved include mitochondrial and endoplasmic reticulum stress, leading to oocyte apoptosis and autophagy, disruption of the cytoskeletal structure, and a reduction in the extrusion rate of the PB1 (Zhai et al., 2018; Santacruz-Márquez et al., 2021; Huang et al., 2022). Animal experiments show the effects of ZnO exposure during gestation on DNA damage in mouse embryonic oocytes and ovarian reserve function in the offspring (Zhai et al., 2018). Notably, ZnO significantly affects cell-oocyte complex expansion; the toxicity of the NPs may be related to the release of ZnO ions and their internalization. Silicon dioxide coating reduces toxicity due to the expansion of the cumulus cell-oocyte complex (Camaioni et al., 2021).

#### 3.4.2 Parabens

Parabens (PBs) are a family of alkyl esters, classified as methylparaben (MePB), ethylparaben (EP), propylparaben (PP), isobutylparaben (IBP), and butylparaben (BP) based on their alkyl chain, and are extensively used in cosmetics, food, and pharmaceuticals (Jeong et al., 2020).

MePB is primarily used in food, personal care, and baby care. A study of *in vitro* culture of porcine oocytes discovered that oocyte maturation was reduced; however, viability was not affected with a 50% lethal concentration (LC50) of 2028.38 μM and a 50% maturation inhibitory concentration (MIC50) of 780.31 μM (Barajas–Salinas et al., 2021). It inhibits oocyte maturation, possibly by inhibiting cumulus cell expansion and altering oocyte morphology (Barajas-Salinas et al., 2021).

IBP is extensively used in personal care products and cosmetics. *In vitro* studies in porcine oocytes showed that low concentrations (200 μM) of IBP significantly reduced the rate of PB1 expulsion but had no significant effect on oocyte expansion; however, high concentrations (400 μM) of IBP significantly affected both. Mechanistically, IBP-treated oocytes showed abnormal spindle bodies, chromosomal misalignment, abnormally distributed actin filaments, elevated levels of H3K9me3 and H3K27me3, and significantly increased oocyte ROS levels and apoptosis rates (Meng et al., 2020).

Additionally, BP is a common preservative, and *in vitro* oocyte cultures present with suppressed meiosis and reduced oocyte expansion, elevated ROS levels, abnormal mitochondrial distribution and function, DNA damage, apoptosis, autophagy, γ-H2AX, annexin V positivity, and significantly higher microtubule-associated LC3 expression (Jeong et al., 2020).

#### 3.4.3 Other toxicants in personal care products

In addition to the substances mentioned above, there are ingredients in personal care products, such as antioxidants, stains, and antibacterial agents, which have been investigated and proven to have harmful effects on oocytes. Para-phenylenediamine (PPD), a common component of hair dyes, has significant toxic effects on human health (Dressler and Appelqvist, 2006). In mice, abnormal oocyte development and reduced fertilization ability with irregular distribution or absence of Juno proteins have been observed. PPD may affect spindle morphology and chromosome aggregation in oocytes and may disrupt oocyte mitochondrial function, promote oxidative stress, and induce early apoptosis (Wang X. et al., 2022). Propyl gallate (PG), an antioxidant commonly used in personal care products, induces DNA damage and oxidative stress, affecting oocyte proliferation and development. Additionally, PG exposure increased histone methylation (H3K27me2 and H3K27me3) in oocytes (Yang et al., 2023).

Organic ultraviolet filters, including oxybenzone (OBZ), benzophenone-3 (BP3), octocrylene (OCL), etc., which are found in human breast milk, amniotic fluid, urine, and blood plasma, are extensively used in personal care products (Ruszkiewicz et al., 2017). They inhibit meiosis and development in animal oocytes by promoting mitochondrial stress and affecting the normal function of the cytoskeleton (Jin et al., 2021; Chang et al., 2022; Tao et al., 2023).

#### 3.4.4 Pharmaceuticals

As environmental toxicants, which have received increasing attention in recent years, PPCPs are derived from various prescription and over-the-counter drugs, antibiotics, hormones, and their metabolites (Qin et al., 2023). Few studies have investigated the effects of these pharmaceuticals on oocytes. In vitro-fertilized women of childbearing age were analyzed for oocyte maturation and embryo quality, and it was concluded that ibuprofen had no effect on oocyte or embryo quality (Schwartz and Burkard, 2020). However, negative effects of hormones on oogenesis have been reported. For example, 17α-ethynylestradiol (EE2) can reduce the abundance of Juno protein and increase ROS levels to promote oocyte apoptosis, which can be inhibited by melatonin (Dai et al., 2020).

### 3.5 Food toxicants

Reproductive toxicity is present in foods; some toxicants, such as mycotoxins, sprouted potatoes, and arecoline, are often ignored.

#### 3.5.1 Mycotoxins

Fusarium mycotoxins include deoxynivalenol (DON), fumonisin B1 (FB1), nivalenol (NIV), beauvericin (BEA), and zearalenone (ZEN); they are primarily produced by fusarium mycotoxins and are commonly detected in agricultural staples such as corn, wheat, oats, barley, peanuts, peas, and many oil grains. They are mainly produced on farmland and may be recontaminated during storage (Hou et al., 2014; Han et al., 2016; Schoevers et al., 2016; Wang et al., 2021a; 2021b). Mycotoxins significantly inhibit the maturation of porcine oocytes by reducing p-MAPK protein levels and disrupting meiosis, delaying cell cycle progression. DON treatment of porcine oocytes with upregulated LC3 protein expression and abnormal Lamp2, LC3, and mTOR mRNA expression induced autophagy and apoptosis. DON exposure increases oocyte DNA methylation by altering DNMT3A mRNA levels. Additionally, histone methylation levels were altered, with increased H3K27me3 and H3K4me2 proteins and related methyltransferase gene mRNA levels (Han et al., 2016). Notably, failure of PB1 extrusion and organelle dysfunction were discovered, with mycotoxin causing impaired PB1 expulsion at concentrations of 30–50 μM and toxic effects on embryos, oocytes, and cumulus cells at concentrations above 50 μM (Schoevers et al., 2016; Wang et al., 2021b). ZEN, a metabolite of Fusarium mycotoxin, acts as a xenoestrogen and is considered cytotoxic, histotoxic, and genotoxic (Hou et al., 2015). ZEN-treated oocytes showed reduced *in vitro* culture maturation rates, impaired mitochondrial membrane potential, and disrupted Golgi apparatus function. In porcine oocytes, ZEN exposure altered ER stress levels, and GRP78 expression was reduced (Hou et al., 2015; Wang Y. et al., 2022).

Ochratoxin A (OTA) is a fungal toxin primarily produced by Aspergillus and Penicillium and is commonly detected in pulses, cereals, grains, spices, and processed products (Huang and Chan, 2016). OTA reduces oocyte quality and quantity by disrupting oocyte meiosis and development, spindle and chromosome arrangement, and extrusion rate of the PB1 (Jia et al., 2020). In addition, OTA induces oxidative stress by inducing the accumulation of ROS during meiosis and the depletion of antioxidants, leading to oocyte apoptosis. Notably, epigenetic modifications in mouse oocytes were affected, as evidenced by the altered levels of 5mC, the global DNA hydroxymethylation (5hmC), H3K9ac, and H3K9me3 (Huang and Chan, 2016; Jia et al., 2020); melatonin has a protective impact against OTA exposure (Lan et al., 2020).

#### 3.5.2 Other food toxicants

4-Methylimidazole (4-MI) is a simple, nitrogen-containing heterocyclic compound. It is commonly used to produce baked goods, soups, beer, and soft drinks. It affects the quality of oocytes by affecting their meiotic ability and fertility. Its toxic effects include mitochondrial dysfunction and meiosis-related cytoskeletal abnormalities (Lu et al., 2022). Solanine is a natural toxin in potato sprouts and often in long-stored potatoes. It was discovered that in porcine oocytes cultured *in vitro*, α-solanine (10 μM) interfered with meiotic progression, increased autophagy-related genes (*LC3*, *ATG7*, and *LAMP2*) and apoptosis-related genes (*BAX* and *CASP3*), and significantly increased the levels of H3K36me3 and H3K27me3 compared to the untreated group (Lin et al., 2018). Arecoline, the primary bioactive substance extracted from betel nuts, affects actin filament kinetics, spindle assembly, and microtubule attachment stability in mouse oocytes, leading to aneuploidy and meiotic arrest. In addition, arecoline treatment disrupts mitochondrial distribution, reduces ATP production, increases oxidative stress, and ultimately induces oocyte apoptosis; metformin has been shown to reverse betaine toxicity (Li et al., 2020).

### 3.6 Fluoride

Fluoride is used globally as an emulsifier in cleaning products, pesticides, food containers, shampoos, toothpaste, etc. (Renner, 2001). A study showed that women with high levels of perfluorinated compounds in their follicular fluid had lower fertilization rates when undergoing *in vitro* fertilization and embryo transplantation (IVF-ET) (Governini et al., 2011).

Exposure to fluoride salt blocks meiosis in porcine oocytes, interferes with spindle dynamics, and increases aneuploidy. It interferes with oocyte mitochondrial function, triggers the DNA damage response, and induces early apoptosis (Liang et al., 2017). In addition, fluoride salt intervention reduced the expression of oocyte-specific genes involved in oocyte growth and induced acrosome response in a dose-dependent manner. Fluorine treatment resulted in lower levels of H3K9ac and H3K18ac in the experimental group than in the control group (Liang et al., 2016).

Perfluorinated compounds are important industrial products extensively used in agricultural activities, the textile industry, food packaging, and cosmetics (Wei et al., 2021). Perfluorodecanoic acid is a synthetic perfluorinated compound that has a significant negative effect on oocyte survival (LC50 = 7.8 μM) and maturation (IM50 = 3.8 μM) by disrupting oocyte calcium homeostasis and gap-junctional intercellular communication (Domínguez et al., 2019). Perfluorohexane sulfonate had a moderate gap-junctional lethal concentration (LC50 = 39.1 μM) of cytotoxicity and inhibited oocyte maturation (MIC50 = 91.68 μM), and had a toxic effect on intercellular communication (Martínez–Quezada et al., 2021). Perfluorooctane sulfonate, a surfactant extensively used in agriculture, causes cytoskeletal abnormalities in oocytes, disrupts mitochondrial function, induces oxidative stress, and triggers early apoptosis. Studies have shown that 600 μM perfluorooctane sulfonate significantly reduces the polar body extrusion rate in mouse oocytes (Wei et al., 2021).

In general, fluorinated compounds have significant reproductive toxicity in animal studies and have been validated in observational studies in humans, although the number of cases is small. However, this evidence indicates that fluorinated compounds should be avoided, especially in mother and child products, food, and personal care products.

## 4 Concluding remarks and future directions

In summary, we have provided an overview of the effects of environmental toxicants on oogenesis and female fertility; this suggests that exposure to heavy metals, cigarette smoke, and agrochemicals can have detrimental effects on oocyte multiplication, growth, and maturation. Several measures can be used to mitigate the effects of environmental toxicants on reproductive health. The primary step involves minimizing or eliminating exposure to toxicants by identifying and avoiding specific sources known to contribute to reproductive toxicity, such as chemicals, pollutants, or occupational hazards. Detoxification and antioxidant strategies help remove toxins from the body and enhance reproductive health. Drinking abundant water and consuming a healthy diet can accelerate liver detoxification. Some antioxidants, including blueberry extracts, glutathione, and melatonin, have been reported to be beneficial for reproductive health. Additionally, consuming foods that support liver function, such as cruciferous vegetables (broccoli, kale, and cabbage), may be beneficial. Guidance from healthcare professionals specializing in reproductive health or environmental medicine is crucial, as they can provide personalized recommendations and suggest targeted therapies or interventions based on an individual’s specific circumstances.

The experimental animal models used in the original studies were primarily rats, pigs, ewes, sheep, and zebrafish. These experimental approaches are limited in their ability to accurately restore the concentration distribution and metabolic processes of environmental toxicants in the human body, which may result in higher doses that amplify toxic effects or inaccurate dose control due to inter-species differences in body size. Furthermore, ethical and feasibility considerations have limited the use of human oocytes as experimental subjects. Despite recent advances in 3D culture and organoid technologies, their application in studying environmental toxins in oocytes remains limited. Therefore, future studies should incorporate models that closely resemble the structure and physiological functions of the human ovary.

Raising awareness of the potential risks of toxin exposure is crucial for promoting public health and preventing adverse reproductive outcomes. To effectively mitigate these risks, it is essential to understand the mechanisms underlying the effects of environmental toxins on reproductive health, which includes the identification of biomarkers that can accurately assess exposure levels and predict reproductive outcomes. Further research is necessary to develop effective strategies for safeguarding the reproductive health of women and their offspring. By advancing our knowledge of the impact of environmental toxins on reproductive health, proactive steps can be taken toward reducing exposure levels and improving overall reproductive outcomes.

Effective prevention and intervention strategies may involve reducing exposure levels and fertility damage by regulating industrial practices or novel treatments for infertility or miscarriages. Furthermore, it is important to provide special protection for women with occupational exposure to the reproductive hazards of environmental toxins, and women of childbearing age should be aware that exposure to environmental toxins can cause damage to oogenesis and the female reproductive system; consequently, safety measures may involve lifestyle changes, such as smoking cessation or dietary modifications. Regarding alleviating exposure to environmental toxins, foods and supplements with antioxidant potential may be effective in protecting the development of oocytes; however, the current evidence is mainly from animal studies, which do not provide significant confidence for their efficacy in humans.

Overall, this study provides evidence for the deleterious effects of environmental toxins on female reproductive health. Further research is warranted to better understand the mechanisms underlying toxicity and the potential protective strategies for oogenesis.

## Figures and Tables

**Table:** 

**4-MI**	4-methylimidazole
**ACE**	Acetamiprid
**As**	Arsenic
**ATP**	Adenosine triphosphate
**AZO**	Azoxystrobin
**BaP**	Benzo(a)pyrene
**BEA**	Beauvericin
**BHPF**	Fuorene-9-bisphenol
**BP**	Butylparaben
**BP3**	Benzophenone-3
**BPA**	Bisphenol A
**BPAF**	Bisphenol AF
**BPS**	Isphenol S
**Cd**	Cadmium
**CF**	Carbofuran
**CPF**	Chlorpyrifos
**Cr**	Chromium
**CrVI**	Hexavalent chromium
**Cu**	Copper
**DBP**	Dibutyl benzodicarboxylate
**DEHP**	Di(2-ethylhexyl) phthalate
**DON**	Deoxynivalenol
**DZN**	Diazinon
**EE2**	17α-ethynylestradiol
**EP**	Ethylparaben
**ER**	Estrogen receptor
**FB1**	Fumonisin B1
**Fe**	Iron
**FPN**	Fipronil
**Hg**	Mercury
**IBP**	Isobutylparaben
**IC50**	Inhibition concentration 50
**IVF**	*In vitro* fertilization
**IVF-ET**	*In vitro* fertilization and embryo transplantation
**LC3**	Protein light chain 3
**LC50**	50% Lethal concentration
**MePB**	Methylparaben
**MIC50**	50% Maturation inhibitory concentration
**MII**	Metaphase II
**miRNAs**	microRNAs
**MNZ**	Dexamethasone zinc
**MP**	Methyl parathion
**MPF**	Maturation promoting factor
**MTOCs**	Microtubule organizing centers
**MTs**	Microtubules
**MXC**	Methoxychlor
**NIV**	Nivalenol
**NNA**	1-(N-methyl-N-nitrosamino)1-(3-pyridinyl)-4-butanal
**NPs**	Nanoparticles
**OBZ**	Oxybenzone
**OCL**	Octocrylene
**OTA**	Ochratoxin A
**PAHs**	Polycyclic aromatic hydrocarbons
**Pb**	lead
**PB1**	First polar body
**PBs**	Parabens
**PCBs**	Polychlorinated biphenyls
**PCOS**	Polycystic ovary syndrome
**PG**	Propyl gallate
**PNMC**	3-methyl-4-nitrophenol
**PP**	Propylparaben
**PPCPs**	Pharmaceuticals and personal care products
**PPD**	Para-phenylenediamine
**PR**	Progesterone receptor
**ROS**	Reactive oxygen species
**ST**	Smokeless tobacco
**TBTO**	Tributyltin oxide
**THS**	Third-hand smoke
**ZEN**	Zearalenone
**Zn**	Zinc
**ZnO NPs**	Zinc oxide nanoparticles

## References

[B1] Altunkaynak B. Z. Akgül N. Yahyazadeh A. Altunkaynak M. E. Turkmen A. P. Akgül H. M. (2016). Effect of mercury vapor inhalation on rat ovary: Stereology and histopathology. J. Obstet. Gynaecol. Res. 42, 410–416. 10.1111/jog.12911 26787318

[B2] Barajas-Salinas A. Ducolomb Y. Betancourt M. Núñez-Macías E. López A. Barraza J. (2021). Effects of methylparaben on *in vitro* maturation of porcine oocytes. J. Appl. Toxicol. 41, 330–337. 10.1002/jat.4045 32767590

[B3] Bloom M. S. Kim D. Vom Saal F. S. Taylor J. A. Cheng G. Lamb J. D. (2011). Bisphenol A exposure reduces the estradiol response to gonadotropin stimulation during *in vitro* fertilization. Fertil. Steril. 96, 672–677. 10.1016/j.fertnstert.2011.06.063 21813122 PMC3168558

[B4] Bolden A. L. Rochester J. R. Schultz K. Kwiatkowski C. F. (2017). Polycyclic aromatic hydrocarbons and female reproductive health: A scoping review. Reprod. Toxicol. 73, 61–74. 10.1016/j.reprotox.2017.07.012 28739294

[B5] Bonilla E. Hernández F. Cortés L. Mendoza M. Mejía J. Carrillo E. (2008). Effects of the insecticides malathion and diazinon on the early oogenesis in mice*in vitro* . Environ. Toxicol. 23, 240–245. 10.1002/tox.20332 18214912

[B6] Budani M. C. Carletti E. Tiboni G. M. (2017). Cigarette smoke is associated with altered expression of antioxidant enzymes in granulosa cells from women undergoing *in vitro* fertilization. Zygote 25, 296–303. 10.1017/S0967199417000132 28635583

[B7] Budani M. C. D’Aurora M. Stuppia L. Gatta V. Tiboni G. M. (2019). Whole‐body exposure to cigarette smoke alters oocyte miRNAs expression in C57BL/6 mice. Mol. Reprod. Dev. 86, 1741–1757. 10.1002/mrd.23267 31512311

[B8] Bukovsky A. Caudle M. R. Svetlikova M. Wimalasena J. Ayala M. E. Dominguez R. (2005). Oogenesis in adult mammals, including humans: A review. Endocr 26, 301–316. 10.1385/ENDO:26:3:301 16034186

[B9] Butts C. D. Bloom M. S. McGough A. Lenhart N. Wong R. Mok-Lin E. (2021). Toxic elements in follicular fluid adversely influence the likelihood of pregnancy and live birth in women undergoing IVF. Hum. Reprod. Open 2021, hoab023. 10.1093/hropen/hoab023 34337160 PMC8318822

[B10] Camaioni A. Massimiani M. Lacconi V. Magrini A. Salustri A. Sotiriou G. A. (2021). Silica encapsulation of ZnO nanoparticles reduces their toxicity for cumulus cell-oocyte-complex expansion. Part Fibre Toxicol. 18, 33. 10.1186/s12989-021-00424-z 34479598 PMC8414698

[B11] Camlin N. J. Sobinoff A. P. Sutherland J. M. Beckett E. L. Jarnicki A. G. Vanders R. L. (2016). Maternal smoke exposure impairs the long-term fertility of female offspring in a murine model. Biol. Reproduction 94, 39. 10.1095/biolreprod.115.135848 26764348

[B12] Cao M. Wang Y. Yang F. Li J. Qin X. (2021). Melatonin rescues the reproductive toxicity of low‐dose glyphosate‐based herbicide during mouse oocyte maturation via the GPER signaling pathway. J. Pineal Res. 70, e12718. 10.1111/jpi.12718 33503294

[B13] Cardoso-Jaime V. Broderick N. A. Maya-Maldonado K. (2022). Metal ions in insect reproduction: A crosstalk between reproductive physiology and immunity. Curr. Opin. Insect Sci. 52, 100924. 10.1016/j.cois.2022.100924 35483647 PMC9357134

[B14] Carson S. A. Kallen A. N. (2021). Diagnosis and management of infertility: A review. JAMA 326, 65–76. 10.1001/jama.2021.4788 34228062 PMC9302705

[B15] Caserta D. Bordi G. Ciardo F. Marci R. La Rocca C. Tait S. (2013). The influence of endocrine disruptors in a selected population of infertile women. Gynecol. Endocrinol. 29, 444–447. 10.3109/09513590.2012.758702 23347089

[B16] Chandravanshi L. P. Gupta R. Shukla R. K. (2018). Developmental neurotoxicity of arsenic: Involvement of oxidative stress and mitochondrial functions. Biol. Trace Elem. Res. 186, 185–198. 10.1007/s12011-018-1286-1 29502250

[B17] Chang H. Li J. Zhang C. Qian W. (2022). Octocrylene exposure impairs mouse oocyte quality by inducing spindle defects and mitochondria dysfunction. Toxicology 479, 153306. 10.1016/j.tox.2022.153306 36049589

[B18] Chen J. Cui Z. Qiu Y. Zhang X. Chen F. Wang H. (2021). Exposure to copper compromises the maturational competency of porcine oocytes by impairing mitochondrial function. Front. Cell. Dev. Biol. 9, 678665. 10.3389/fcell.2021.678665 34150773 PMC8212058

[B19] Cheng J. Mi P. Li Y. Lu Y. Sun F. (2022). Melatonin prevents oocyte deterioration due to cotinine exposure in mice. Biol. Reproduction 107, 635–649. 10.1093/biolre/ioac043 35191979

[B20] Cheng S.-F. Qin X.-S. Han Z.-L. Sun X.-F. Feng Y.-N. Yang F. (2018). Nicotine exposure impairs germ cell development in human fetal ovaries cultured *in vitro* . Aging 10, 1556–1574. 10.18632/aging.101492 30001218 PMC6075447

[B21] Cheng Y. Zhang J. Wu T. Jiang X. Jia H. Qing S. (2019). Reproductive toxicity of acute Cd exposure in mouse: Resulting in oocyte defects and decreased female fertility. Toxicol. Appl. Pharmacol. 379, 114684. 10.1016/j.taap.2019.114684 31325558

[B22] Choi H. Lee J. Yoon J. D. Hwang S.-U. Cai L. Kim M. (2021). The effect of copper supplementation on *in vitro* maturation of porcine cumulus-oocyte complexes and subsequent developmental competence after parthenogenetic activation. Theriogenology 164, 84–92. 10.1016/j.theriogenology.2021.01.009 33567360

[B23] Choi H. Oh D. Kim M. Cai L. Lee J. Kim E. (2022). Copper deficiency affects the developmental competence of porcine oocytes matured *in vitro* . Front. Cell. Dev. Biol. 10, 993030. 10.3389/fcell.2022.993030 36158185 PMC9490373

[B24] Cinar O. Semiz O. Can A. (2015). Carbofuran alters centrosome and spindle organization, and delays cell division in oocytes and mitotic cells. Toxicol. Sci. 144, 298–306. 10.1093/toxsci/kfu317 25564422

[B25] Coticchio G. Dal Canto M. Mignini Renzini M. Guglielmo M. C. Brambillasca F. Turchi D. (2015). Oocyte maturation: Gamete-somatic cells interactions, meiotic resumption, cytoskeletal dynamics and cytoplasmic reorganization. Hum. Reprod. Update 21, 427–454. 10.1093/humupd/dmv011 25744083

[B26] Dai X. Qiu L. Zhao B. Gao Y. Mu Y. Chu Z. (2020). Melatonin ameliorates the fertilization capacity of oocytes exposed to 17α-ethynylestradiol. Reprod. Toxicol. 93, 61–67. 10.1016/j.reprotox.2020.01.004 31931096

[B27] Dickerson E. H. Sathyapalan T. Knight R. Maguiness S. M. Killick S. R. Robinson J. (2011). Endocrine disruptor & nutritional effects of heavy metals in ovarian hyperstimulation. J. Assist. Reprod. Genet. 28, 1223–1228. 10.1007/s10815-011-9652-3 22071884 PMC3241832

[B28] Ding Z.-M. Hua L.-P. Ahmad M. J. Safdar M. Chen F. Wang Y.-S. (2020). Diethylstilbestrol exposure disrupts mouse oocyte meiotic maturation *in vitro* through affecting spindle assembly and chromosome alignment. Chemosphere 249, 126182. 10.1016/j.chemosphere.2020.126182 32078850

[B29] Ding Z.-M. Jiao X.-F. Wu D. Zhang J.-Y. Chen F. Wang Y.-S. (2017). Bisphenol AF negatively affects oocyte maturation of mouse *in vitro* through increasing oxidative stress and DNA damage. Chemico-Biological Interact. 278, 222–229. 10.1016/j.cbi.2017.10.030 29102535

[B30] Dinisri I. Kodikara S. Prasadani M. Pathirana I. Rathnayake C. Alexander B. (2021). Impairment of caprine oocyte maturation *in vitro* and alteration of granulosa cells functions by widely used fungicide mancozeb. Trop. Anim. Health Prod. 53, 406. 10.1007/s11250-021-02854-5 34287714

[B31] Domínguez A. Salazar Z. Betancourt M. Ducolomb Y. Casas E. Fernández F. (2019). Effect of perfluorodecanoic acid on pig oocyte viability, intracellular calcium levels and gap junction intercellular communication during oocyte maturation *in vitro* . Toxicol. Vitro 58, 224–229. 10.1016/j.tiv.2019.03.041 30946969

[B32] Dong F. Li J. Lei W.-L. Wang F. Wang Y. Ouyang Y.-C. (2020). Chronic cadmium exposure causes oocyte meiotic arrest by disrupting spindle assembly checkpoint and maturation promoting factor. Reprod. Toxicol. 96, 141–149. 10.1016/j.reprotox.2020.06.009 32574675

[B33] Dressler W. E. Appelqvist T. (2006). Plasma/blood pharmacokinetics and metabolism after dermal exposure to para-aminophenol or para-phenylenediamine. Food Chem. Toxicol. 44, 371–379. 10.1016/j.fct.2005.08.009 16239057

[B34] Ehrlich S. Williams P. L. Missmer S. A. Flaws J. A. Berry K. F. Calafat A. M. (2012a). Urinary bisphenol A concentrations and implantation failure among women undergoing *in vitro* fertilization. Environ. Health Perspect. 120, 978–983. 10.1289/ehp.1104307 22484414 PMC3404656

[B35] Ehrlich S. Williams P. L. Missmer S. A. Flaws J. A. Ye X. Calafat A. M. (2012b). Urinary bisphenol A concentrations and early reproductive health outcomes among women undergoing IVF. Hum. Reprod. 27, 3583–3592. 10.1093/humrep/des328 23014629 PMC3501244

[B36] Einaudi L. Courbiere B. Tassistro V. Prevot C. Sari-Minodier I. Orsiere T. (2014). *In vivo* exposure to benzo(a)pyrene induces significant DNA damage in mouse oocytes and cumulus cells. Hum. Reprod. 29, 548–554. 10.1093/humrep/det439 24327538

[B37] El-Sharkawy E. E. Kames A. O. G. Sayed S. M. Nisr N. A. E. L. Wahba N. M. Elsherif W. M. (2014). The ameliorative effect of propolis against methoxychlor induced ovarian toxicity in rat. Exp. Toxicol. Pathology 66, 415–421. 10.1016/j.etp.2014.06.003 25034310

[B38] Esmaiel S. Tahereh H. Noreddin N.-M. S. Massood E. (2019). Mancozeb exposure during development and lactation periods results in decreased oocyte maturation, fertilization rates, and implantation in the first-generation mice pups: Protective effect of vitamins E and C. Toxicol. Ind. Health 35, 714–725. 10.1177/0748233719890965 31818241

[B39] Ferris J. Mahboubi K. MacLusky N. King W. A. Favetta L. A. (2016). BPA exposure during *in vitro* oocyte maturation results in dose-dependent alterations to embryo development rates, apoptosis rate, sex ratio and gene expression. Reprod. Toxicol. 59, 128–138. 10.1016/j.reprotox.2015.12.002 26686065

[B40] Fitzgerald A. C. Peyton C. Dong J. Thomas P. (2015). Bisphenol A and related alkylphenols exert nongenomic estrogenic actions through a G protein-coupled estrogen receptor 1 (Gper)/Epidermal growth factor receptor (egfr) pathway to inhibit meiotic maturation of zebrafish oocytes. 10.1095/biolreprod.115.132316 PMC4712694 26490843

[B41] Flores D. Souza V. Betancourt M. Teteltitla M. González-Márquez H. Casas E. (2017). Oxidative stress as a damage mechanism in porcine cumulus-oocyte complexes exposed to malathion during *in vitro* maturation: FLORES et al. Environ. Toxicol. 32, 1669–1678. 10.1002/tox.22384 28185390

[B42] Foster W. G. Hughes C. L. (2011). Gene expression in oogenesis and implications for transgenerational effects of environmental toxicants. Biol. Reprod. 84, 2–4. 10.1095/biolreprod.110.088260 20864646

[B43] Fowler P. A. Childs A. J. Courant F. MacKenzie A. Rhind S. M. Antignac J.-P. (2014). *In utero* exposure to cigarette smoke dysregulates human fetal ovarian developmental signalling. Hum. Reprod. 29, 1471–1489. 10.1093/humrep/deu117 24847019

[B44] Fréour T. Dessolle L. Lammers J. Lattes S. Barrière P. (2013). Comparison of embryo morphokinetics after *in vitro* fertilization-intracytoplasmic sperm injection in smoking and nonsmoking women. Fertil. Steril. 99, 1944–1950. 10.1016/j.fertnstert.2013.01.136 23465820

[B45] Fréour T. Massart P. García D. Vassena R. Rodríguez A. (2018). Revisiting the association between smoking and female fertility using the oocyte donation model. Reprod. Biomed. Online 37, 564–572. 10.1016/j.rbmo.2018.08.018 30366838

[B46] Gai Y. Zhang M.-Y. Ji P.-Y. You R.-J. Ge Z.-J. Shen W. (2022). Melatonin improves meiosis maturation against diazinon exposure in mouse oocytes. Life Sci. 301, 120611. 10.1016/j.lfs.2022.120611 35526594

[B47] Gao W. Zhang C. Li B. Oh J. S. (2022). Azoxystrobin exposure impairs meiotic maturation by disturbing spindle formation in mouse oocytes. Front. Cell. Dev. Biol. 10, 1053654. 10.3389/fcell.2022.1053654 36531942 PMC9755494

[B48] GBD 2019 Demographics Collaborators (2020). Global age-sex-specific fertility, mortality, healthy life expectancy (HALE), and population estimates in 204 countries and territories, 1950-2019: A comprehensive demographic analysis for the global burden of disease study 2019. Lancet 396, 1160–1203. 10.1016/S0140-6736(20)30977-6 33069325 PMC7566045

[B49] Ge C. Ye J. Wang Q. Zhang C. Yang J.-M. Qian G. (2012). Polycyclic aromatic hydrocarbons suppress meiosis in primordial germ cells via the AHR signaling pathway. Toxicol. Lett. 210, 285–292. 10.1016/j.toxlet.2012.02.014 22387805

[B50] Gely-Pernot A. Saci S. Kernanec P.-Y. Hao C. Giton F. Kervarrec C. (2017). Embryonic exposure to the widely-used herbicide atrazine disrupts meiosis and normal follicle formation in female mice. Sci. Rep. 7, 3526. 10.1038/s41598-017-03738-1 28615648 PMC5471253

[B51] Gonsioroski A. V. Aquino A. M. Alonso-Costa L. G. Barbisan L. F. Scarano W. R. Flaws J. A. (2022). Multigenerational effects of an environmentally relevant phthalate mixture on reproductive parameters and ovarian miRNA expression in female rats. Toxicol. Sci. 189, 91–106. 10.1093/toxsci/kfac066 35762964 PMC9801715

[B52] Gosden R. G. (2002). Oogenesis as a foundation for embryogenesis. Mol. Cell. Endocrinol. 186, 149–153. 10.1016/S0303-7207(01)00683-9 11900888

[B53] Governini L. Orvieto R. Guerranti C. Gambera L. De Leo V. Piomboni P. (2011). The impact of environmental exposure to perfluorinated compounds on oocyte fertilization capacity. J. Assist. Reprod. Genet. 28, 415–418. 10.1007/s10815-011-9548-2 21344220 PMC3151367

[B54] Guo J. Guo W. Zhang T. Zheng Y. Han B. Zhang Z. (2022). Gestational exposure to phenanthrene induces follicular atresia and endocrine dyscrasia in F1 adult female. Ecotoxicol. Environ. Saf. 232, 113291. 10.1016/j.ecoenv.2022.113291 35158277

[B55] Han J. Wang Q.-C. Zhu C.-C. Liu J. Zhang Y. Cui X.-S. (2016). Deoxynivalenol exposure induces autophagy/apoptosis and epigenetic modification changes during porcine oocyte maturation. Toxicol. Appl. Pharmacol. 300, 70–76. 10.1016/j.taap.2016.03.006 26988607

[B56] Han Y. Zhang S. Wang Z. Zhang L. Zhang F. Sun F. (2018). Toxicological effects of 3-methyl-4-nitrophenol on mouse ovarian and testicular cell proliferation, apoptosis and oocyte maturation. Reprod. Toxicol. 82, 94–102. 10.1016/j.reprotox.2018.10.005 30352283

[B57] He D. Xu Y. Hou L. Wang J. Yang S. Wang Y. (2022a). Toxic effects of methomyl on mouse oocytes and its possible mechanisms. Zygote 30, 358–364. 10.1017/S0967199421000782 34676817

[B58] He Q.-K. Xu C.-L. Li Y.-P. Xu Z.-R. Luo Y.-S. Zhao S.-C. (2022b). Captan exposure disrupts ovarian homeostasis and affects oocytes quality via mitochondrial dysfunction induced apoptosis. Chemosphere 286, 131625. 10.1016/j.chemosphere.2021.131625 34303901

[B59] He Y.-T. Wang W. Shen W. Sun Q.-Y. Yin S. (2019a). Melatonin protects against Fenoxaprop-ethyl exposure-induced meiotic defects in mouse oocytes. Toxicology 425, 152241. 10.1016/j.tox.2019.152241 31265864

[B60] He Y.-T. Yang L.-L. Zhao Y. Shen W. Yin S. Sun Q.-Y. (2019b). Fenoxaprop-ethyl affects mouse oocyte quality and the underlying mechanisms: Fenoxaprop-ethyl affects mouse oocyte quality. Pest. Manag. Sci. 75, 844–851. 10.1002/ps.5190 30152098

[B61] Heo G. Sun M.-H. Jiang W.-J. Li X.-H. Lee S.-H. Guo J. (2022). Rotenone causes mitochondrial dysfunction and prevents maturation in porcine oocytes. PLoS ONE 17, e0277477. 10.1371/journal.pone.0277477 36441709 PMC9704683

[B62] Hou Y.-J. Xiong B. Zheng W.-J. Duan X. Cui X.-S. Kim N.-H. (2014). Oocyte quality in mice is affected by a mycotoxin-contaminated diet: Mycotoxins Affect Mouse Oocyte Quality. Environ. Mol. Mutagen. 55, 354–362. 10.1002/em.21833 24288346

[B63] Hou Y.-J. Zhu C.-C. Xu Y.-X. Cui X.-S. Kim N.-H. Sun S.-C. (2015). Zearalenone exposure affects mouse oocyte meiotic maturation and granulosa cell proliferation: Zen Effects on Oocytes. Environ. Toxicol. 30, 1226–1233. 10.1002/tox.21995 24733567

[B64] Huang C. Wu D. Khan F. A. Wang Y. Xu J. Luo C. (2022). Zinc oxide nanoparticle causes toxicity to the development of mouse oocyte and early embryo. Toxicol. Lett. 358, 48–58. 10.1016/j.toxlet.2022.01.010 35074469

[B65] Huang F.-J. Chan W.-H. (2016). Effects of ochratoxin a on mouse oocyte maturation and fertilization, and apoptosis during fetal development: Effects of Ochratoxin a on Oocyte Maturation and Related Processes. Environ. Toxicol. 31, 724–735. 10.1002/tox.22085 25504763

[B66] Ishikawa S. Hiraga K. Hiradate Y. Tanemura K. (2015). The effects analysis of two neonicotinoid insecticides on *in vitro* maturation of porcine oocytes using hanging drop monoculture method. J. Veterinary Med. Sci. 77, 725–728. 10.1292/jvms.15-0008 PMC4488412 25715671

[B67] Izaguirry A. P. Soares M. B. Vargas L. M. Spiazzi C. C. Dos Santos Brum D. Noremberg S. (2017). Blueberry (Vaccinium ashei Reade) extract ameliorates ovarian damage induced by subchronic cadmium exposure in mice: Potential δ-ALA-D involvement. Environ. Toxicol. 32, 188–196. 10.1002/tox.22225 26663770

[B68] Jennings P. C. Merriman J. A. Beckett E. L. Hansbro P. M. Jones K. T. (2011). Increased zona pellucida thickness and meiotic spindle disruption in oocytes from cigarette smoking mice. Hum. Reprod. 26, 878–884. 10.1093/humrep/deq393 21233109

[B69] Jeong P.-S. Lee S. Park S.-H. Kim M. J. Kang H.-G. Nanjidsuren T. (2020). Butylparaben is toxic to porcine oocyte maturation and subsequent embryonic development following *in vitro* fertilization. IJMS 21, 3692. 10.3390/ijms21103692 32456265 PMC7279239

[B70] Jia H. Jia C. An Q. Cheng Y. Jiang X. Xu Y. (2020). Ochratoxin A exposure causes meiotic failure and oocyte deterioration in mice. Theriogenology 148, 236–248. 10.1016/j.theriogenology.2019.11.005 31735432

[B71] Jia Z.-Z. Zhang J.-W. Zhou D. Xu D.-Q. Feng X.-Z. (2019b). Deltamethrin exposure induces oxidative stress and affects meiotic maturation in mouse oocyte. Chemosphere 223, 704–713. 10.1016/j.chemosphere.2019.02.092 30802836

[B72] Jia Z. Wang H. Feng Z. Zhang S. Wang L. Zhang J. (2019a). Fluorene-9-bisphenol exposure induces cytotoxicity in mouse oocytes and causes ovarian damage. Ecotoxicol. Environ. Saf. 180, 168–178. 10.1016/j.ecoenv.2019.05.019 31082581

[B73] Jiang X. Xing X. Zhang Y. Zhang C. Wu Y. Chen Y. (2021a). Lead exposure activates the Nrf2/Keap1 pathway, aggravates oxidative stress, and induces reproductive damage in female mice. Ecotoxicol. Environ. Saf. 207, 111231. 10.1016/j.ecoenv.2020.111231 32916527

[B74] Jiang Y. He Y. Li W. Ni J. Li J. Peng L. (2021b). Exposure to chlorpyrifos leads to spindle disorganization and mitochondrial dysfunction of porcine oocytes during *in vitro* maturation. Theriogenology 173, 249–260. 10.1016/j.theriogenology.2021.08.007 34399389

[B75] Jiao X. Ding Z. Meng F. Zhang X. Wang Y. Chen F. (2020). The toxic effects of Fluorene‐9‐bisphenol on porcine oocyte *in vitro* maturation. Environ. Toxicol. 35, 152–158. 10.1002/tox.22851 31696613

[B76] Jin L. Zhu H.-Y. Kang X.-J. Lin L.-P. Zhang P.-Y. Tan T. (2021). Melatonin protects against oxybenzone-induced deterioration of mouse oocytes during maturation. Aging 13, 2727–2749. 10.18632/aging.202323 PMC7880374 33373318

[B77] Kandaraki E. Chatzigeorgiou A. Livadas S. Palioura E. Economou F. Koutsilieris M. (2011). Endocrine disruptors and polycystic ovary syndrome (PCOS): Elevated serum levels of bisphenol A in women with PCOS. J. Clin. Endocrinol. Metab. 96, E480–E484. 10.1210/jc.2010-1658 21193545

[B78] Kang H.-G. Jeong P.-S. Kim M. J. Joo Y. E. Gwon M.-A. Jeon S.-B. (2022). Arsenic exposure during porcine oocyte maturation negatively affects embryonic development by triggering oxidative stress-induced mitochondrial dysfunction and apoptosis. Toxicology 480, 153314. 10.1016/j.tox.2022.153314 36084880

[B79] Laldinsangi C. (2022). Toxic effects of smokeless tobacco on female reproductive health: A review. Curr. Res. Toxicol. 3, 100066. 10.1016/j.crtox.2022.100066 35310558 PMC8927787

[B80] Lan M. Zhang Y. Wan X. Pan M.-H. Xu Y. Sun S.-C. (2020). Melatonin ameliorates ochratoxin A-induced oxidative stress and apoptosis in porcine oocytes. Environ. Pollut. 256, 113374. 10.1016/j.envpol.2019.113374 31672358

[B81] Legoff L. Dali O. D’Cruz S. C. Suglia A. Gely-Pernot A. Hémery C. (2019). Ovarian dysfunction following prenatal exposure to an insecticide, chlordecone, associates with altered epigenetic features. Epigenetics Chromatin 12, 29. 10.1186/s13072-019-0276-7 31084621 PMC6515617

[B82] Li A. Ni Z. Zhang J. Cai Z. Kuang Y. Yu C. (2020). Transferrin insufficiency and iron overload in follicular fluid contribute to oocyte dysmaturity in infertile women with advanced endometriosis. Front. Endocrinol. 11, 391. 10.3389/fendo.2020.00391 PMC7317002 32636803

[B83] Li F.-P. Zhou J.-L. Guo A.-W. Liu Y. Zhang F. Xu B.-H. (2019). Di(n-butyl) phthalate exposure impairs meiotic competence and development of mouse oocyte. Environ. Pollut. 246, 597–607. 10.1016/j.envpol.2018.12.077 30605815

[B84] Li M.-H. Wang J.-J. Feng Y.-Q. Liu X. Yan Z.-H. Zhang X.-J. (2023). H3K4me3 as a target of di(2-ethylhexyl) phthalate (DEHP) impairing primordial follicle assembly. Chemosphere 310, 136811. 10.1016/j.chemosphere.2022.136811 36220427

[B85] Li W. Zang C. Yin S. Shen W. Sun Q. Zhao M. (2020). Metformin protects against mouse oocyte apoptosis defects induced by arecoline. Cell. Prolif. 53, e12809. 10.1111/cpr.12809 32557964 PMC7377942

[B86] Liang C. Luo G. Cao Y. Li D. Shen L. Zhang Z. (2022). Environmental thallium exposure and the risk of early embryonic arrest among women undergoing *in vitro* fertilization: Thallium exposure and polymorphisms of mtDNA gene interaction and potential cause exploring. Environ. Sci. Pollut. Res. 29, 62648–62661. 10.1007/s11356-022-19978-2 35411517

[B87] Liang S. Nie Z.-W. Zhao M. Niu Y.-J. Shin K.-T. Cui X.-S. (2017). Sodium fluoride exposure exerts toxic effects on porcine oocyte maturation. Sci. Rep. 7, 17082. 10.1038/s41598-017-17357-3 29213094 PMC5719058

[B88] Liang S. Zhao M.-H. Ock S. A. Kim N.-H. Cui X.-S. (2016). Fluoride impairs oocyte maturation and subsequent embryonic development in mice: Fluoride impairs oocyte maturation and subsequent embryonic development in mice. Environ. Toxicol. 31, 1486–1495. 10.1002/tox.22153 26011085

[B89] Lin T. Oqani R. K. Lee J. E. Kang J. W. Kim S. Y. Cho E. S. (2018). α-Solanine impairs oocyte maturation and quality by inducing autophagy and apoptosis and changing histone modifications in a pig model. Reprod. Toxicol. 75, 96–109. 10.1016/j.reprotox.2017.12.005 29247839

[B90] Liu H. Liu Z. Lu T. Zhang L. Cheng J. Fu X. (2019a). Toxic effects of 1-(N-methyl-N-nitrosamino)-1-(3-pyridinyl)-4-butanal on the maturation and subsequent development of murine oocyte. Ecotoxicol. Environ. Saf. 181, 370–380. 10.1016/j.ecoenv.2019.06.006 31212185

[B91] Liu H. Liu Z. Meng L. Fu X. Hou Y. (2019b). Toxic effects of 1-(N-methyl-N-nitrosamino)-1-(3-pyridinyl)-4-butanal on the reproduction of female mice. Ecotoxicol. Environ. Saf. 183, 109544. 10.1016/j.ecoenv.2019.109544 31400720

[B92] Liu J. Lu X. Wang W. Zhu J. Li Y. Luo L. (2018). Activity of MPF and expression of its related genes in mouse MI oocytes exposed to cadmium. Food Chem. Toxicol. 112, 332–341. 10.1016/j.fct.2017.12.046 29287790

[B93] Liu S.-Z. Wei Z.-F. Meng X.-Q. Han X.-Y. Cheng D. Zhong T. (2016a). Exposure to aroclor-1254 impairs spindle assembly during mouse oocyte maturation. Environ. Toxicol. 31, 1652–1662. 10.1002/tox.22169 26174069

[B94] Liu W.-X. Tan S.-J. Wang Y.-F. Li L. Sun X.-F. Liu J. (2020). Melatonin ameliorates murine fetal oocyte meiotic dysfunction in F1 and F2 offspring caused by nicotine exposure during pregnancy. Environ. Pollut. 263, 114519. 10.1016/j.envpol.2020.114519 32325354

[B95] Liu W.-X. Zhang Y.-J. Wang Y.-F. Klinger F. G. Tan S.-J. Farini D. (2021a). Protective mechanism of luteinizing hormone and follicle-stimulating hormone against nicotine-induced damage of mouse early folliculogenesis. Front. Cell. Dev. Biol. 9, 723388. 10.3389/fcell.2021.723388 34557491 PMC8452944

[B96] Liu Y. He Q.-K. Xu Z.-R. Xu C.-L. Zhao S.-C. Luo Y.-S. (2021b). Thiamethoxam exposure induces endoplasmic reticulum stress and affects ovarian function and oocyte development in mice. J. Agric. Food Chem. 69, 1942–1952. 10.1021/acs.jafc.0c06340 33533595

[B97] Liu Y. Wang Y.-L. Chen M.-H. Zhang Z. Xu B.-H. Liu R. (2016). Methoxychlor exposure induces oxidative stress and affects mouse oocyte meiotic maturation: M ethoxychlor a ffects M ouse O ocyte M aturation. Mol. Reprod. Dev. 83, 768–779. 10.1002/mrd.22683 27434785

[B98] Lu Y. Tang H. Xu J. Sun F. (2022). Toxic effects of 4-methylimidazole on the maturation and fertilization of mouse oocytes. Food Chem. Toxicol. 164, 113051. 10.1016/j.fct.2022.113051 35460824

[B99] MacLennan M. Crichton J. H. Playfoot C. J. Adams I. R. (2015). Oocyte development, meiosis and aneuploidy. Semin. Cell. Dev. Biol. 45, 68–76. 10.1016/j.semcdb.2015.10.005 26454098 PMC4828587

[B100] Mai Z. Lei M. Yu B. Du H. Liu J. (2014). The effects of cigarette smoke extract on ovulation, oocyte morphology and ovarian gene expression in mice. PLoS ONE 9, e95945. 10.1371/journal.pone.0095945 24776817 PMC4002431

[B101] Malott K. F. Leon Parada K. Lee M. Swanson E. Luderer U. (2022). Gestational benzo[a]pyrene exposure destroys F1 ovarian germ cells through mitochondrial apoptosis pathway and diminishes surviving oocyte quality. Toxicol. Sci. 190, 23–40. 10.1093/toxsci/kfac086 35993611 PMC9960072

[B102] Mao T. Han C. Wei B. Zhao L. Zhang Q. Deng R. (2018). Protective effects of quercetin against cadmium chloride-induced oxidative injury in goat sperm and zygotes. Biol. Trace Elem. Res. 185, 344–355. 10.1007/s12011-018-1255-8 29397540

[B103] Martínez-Quezada R. González-Castañeda G. Bahena I. Domínguez A. Domínguez-López P. Casas E. (2021). Effect of perfluorohexane sulfonate on pig oocyte maturation, gap-junctional intercellular communication, mitochondrial membrane potential and DNA damage in cumulus cells *in vitro* . Toxicol. Vitro 70, 105011. 10.1016/j.tiv.2020.105011 33038467

[B104] Meng F. Jiao X. Chen F. Zhang X. Duan Z. Ding Z. (2020). Isobutylparaben negatively affects porcine oocyte maturation through increasing oxidative stress and cytoskeletal abnormalities. Environ. Mol. Mutagen 61, 433–444. 10.1002/em.22356 31922297

[B105] Mirihagalle S. You T. Suh L. Patel C. Gao L. Rattan S. (2019). Prenatal exposure to di-(2-ethylhexyl) phthalate and high-fat diet synergistically disrupts mouse fetal oogenesis and affects folliculogenesis. Biol. Reproduction 100, 1561–1570. 10.1093/biolre/ioz051 PMC7302514 30939196

[B106] Molavi M. Razi M. Cheraghi H. Khorramjouy M. Ostadi A. Gholirad S. (2016). Protective effect of vitamin E on cypermethrin-induced follicular atresia in rat ovary: Evidence for energy dependent mechanism. PMC4959340 27482357

[B107] Mu X. Liao X. Chen X. Li Y. Wang M. Shen C. (2015). DEHP exposure impairs mouse oocyte cyst breakdown and primordial follicle assembly through estrogen receptor-dependent and independent mechanisms. J. Hazard. Mater. 298, 232–240. 10.1016/j.jhazmat.2015.05.052 26073378

[B108] Nair R. Singh V. J. Salian S. R. Kalthur S. G. D’Souza A. S. Shetty P. K. (2014). Methyl parathion inhibits the nuclear maturation, decreases the cytoplasmic quality in oocytes and alters the developmental potential of embryos of Swiss albino mice. Toxicol. Appl. Pharmacol. 279, 338–350. 10.1016/j.taap.2014.07.004 25038315

[B109] Nakano K. Nishio M. Kobayashi N. Hiradate Y. Hoshino Y. Sato E. (2016). Comparison of the effects of BPA and BPAF on oocyte spindle assembly and polar body release in mice. Zygote 24, 172–180. 10.1017/S0967199415000027 25925194

[B110] Nandi S. Gupta P. S. P. Selvaraju S. Roy S. C. Ravindra J. P. (2010). Effects of exposure to heavy metals on viability, maturation, fertilization, and embryonic development of buffalo (Bubalus bubalis) oocytes *in vitro* . Arch. Environ. Contam. Toxicol. 58, 194–204. 10.1007/s00244-009-9342-7 19475365

[B111] Nava-Rivera L. E. Betancourt-Martínez N. D. Lozoya-Martínez R. Carranza-Rosales P. Guzmán-Delgado N. E. Carranza-Torres I. E. (2021). Transgenerational effects in DNA methylation, genotoxicity and reproductive phenotype by chronic arsenic exposure. Sci. Rep. 11, 8276. 10.1038/s41598-021-87677-y 33859283 PMC8050275

[B112] Ommati M. M. Shi X. Li H. Zamiri M. J. Farshad O. Jamshidzadeh A. (2020). The mechanisms of arsenic-induced ovotoxicity, ultrastructural alterations, and autophagic related paths: An enduring developmental study in folliculogenesis of mice. Ecotoxicol. Environ. Saf. 204, 110973. 10.1016/j.ecoenv.2020.110973 32781346

[B113] Ozbakir B. Tulay P. (2020). Does cigarette smoking really have a clinical effect on folliculogenesis and oocyte maturation? Zygote 28, 318–321. 10.1017/S0967199420000155 32338250

[B114] Paixão L. L. Gaspar-Reis R. P. Gonzalez G. P. Santos A. S. Santana A. C. Santos R. M. (2012). Cigarette smoke impairs granulosa cell proliferation and oocyte growth after exposure cessation in young Swiss mice: An experimental study. J. Ovarian Res. 5, 25. 10.1186/1757-2215-5-25 22995067 PMC3489515

[B115] Pan M.-H. Wu Y.-K. Liao B.-Y. Zhang H. Li C. Wang J.-L. (2021). Bisphenol A exposure disrupts organelle distribution and functions during mouse oocyte maturation. Front. Cell. Dev. Biol. 9, 661155. 10.3389/fcell.2021.661155 33834027 PMC8021768

[B116] Patel U. N. Patel U. D. Khadayata A. V. Vaja R. K. Modi C. M. Patel H. B. (2022). Long-term exposure of the binary mixture of cadmium and mercury damages the developed ovary of adult zebrafish. Environ. Sci. Pollut. Res. 29, 44928–44938. 10.1007/s11356-022-18988-4 35138535

[B117] Petr J. Chmelíková E. Žalmanová T. Tůmová L. Kheilová K. Kučerová-Chrpová V. (2013). Pyrethroids cypermethrin, deltamethrin and fenvalerate have different effects on *in vitro* maturation of pig oocytes at different stages of growth. Animal 7, 134–142. 10.1017/S1751731112001140 23031310

[B118] Priya K. Setty M. Babu U. V. Pai K. S. R. (2021). Implications of environmental toxicants on ovarian follicles: How it can adversely affect the female fertility? Environ. Sci. Pollut. Res. 28, 67925–67939. 10.1007/s11356-021-16489-4 PMC8718383 34628616

[B119] Prokešová Š. Ghaibour K. Liška F. Klein P. Fenclová T. Štiavnická M. (2020). Acute low-dose bisphenol S exposure affects mouse oocyte quality. Reprod. Toxicol. 93, 19–27. 10.1016/j.reprotox.2019.12.005 31881267

[B120] Qin H. Liu H. Liu Y. Di S. Bao Y. Zhai Y. (2023). Recent advances in sample preparation and chromatographic analysis of pharmaceuticals and personal care products in environment. TrAC Trends Anal. Chem. 164, 117112. 10.1016/j.trac.2023.117112

[B121] Ren J. Li S. Wang C. Hao Y. Liu Z. Ma Y. (2022). Glutathione protects against the meiotic defects of ovine oocytes induced by arsenic exposure via the inhibition of mitochondrial dysfunctions. Ecotoxicol. Environ. Saf. 230, 113135. 10.1016/j.ecoenv.2021.113135 34979315

[B122] Ren J. Wang B. Li L. Li S. Ma Y. Su L. (2023). Glutathione ameliorates the meiotic defects of copper exposed ovine oocytes via inhibiting the mitochondrial dysfunctions. Ecotoxicol. Environ. Saf. 251, 114530. 10.1016/j.ecoenv.2023.114530 36630773

[B123] Renner R. (2001). Growing concern over perfluorinated chemicals. Environ. Sci. Technol. 35, 154A-160A–160A. 10.1021/es012317k 11348100

[B124] Ruszkiewicz J. A. Pinkas A. Ferrer B. Peres T. V. Tsatsakis A. Aschner M. (2017). Neurotoxic effect of active ingredients in sunscreen products, a contemporary review. Toxicol. Rep. 4, 245–259. 10.1016/j.toxrep.2017.05.006 28959646 PMC5615097

[B125] Rzymski P. Tomczyk K. Rzymski P. Poniedziałek B. Opala T. Wilczak M. (2015). Impact of heavy metals on the female reproductive system. Ann. Agric. Environ. Med. 22, 259–264. 10.5604/12321966.1152077 26094520

[B126] Sadeu J. C. Foster W. G. (2011a). Cigarette smoke condensate exposure delays follicular development and function in a stage-dependent manner. Fertil. Steril. 95, 2410–2417. 10.1016/j.fertnstert.2011.03.072 21514584

[B127] Sadeu J. C. Foster W. G. (2011b). Effect of *in vitro* exposure to benzo[a]pyrene, a component of cigarette smoke, on folliculogenesis, steroidogenesis and oocyte nuclear maturation. Reprod. Toxicol. 31, 402–408. 10.1016/j.reprotox.2010.12.006 21172420

[B128] Saleh A. C. Sabry R. Mastromonaco G. F. Favetta L. A. (2021). BPA and BPS affect the expression of anti-Mullerian hormone (AMH) and its receptor during bovine oocyte maturation and early embryo development. Reprod. Biol. Endocrinol. 19, 119. 10.1186/s12958-021-00773-6 34344364 PMC8330045

[B129] Sánchez F. Smitz J. (2012). Molecular control of oogenesis. Biochimica Biophysica Acta (BBA) - Mol. Basis Dis. 1822, 1896–1912. 10.1016/j.bbadis.2012.05.013 22634430

[B130] Santacruz-Márquez R. González-De Los Santos M. Hernández-Ochoa I. (2021). Ovarian toxicity of nanoparticles. Reprod. Toxicol. 103, 79–95. 10.1016/j.reprotox.2021.06.002 34098047

[B131] Satar D. A. Tap O. Ay M. O. (2015). Electron microscopic examination of the effects of methyl parathion exposure on the ovaries. 26221906

[B132] Schoevers E. J. Santos R. R. Fink-Gremmels J. Roelen B. A. J. (2016). Toxicity of beauvericin on porcine oocyte maturation and preimplantation embryo development. Reprod. Toxicol. 65, 159–169. 10.1016/j.reprotox.2016.07.017 27474255

[B133] Schultz R. M. Letourneau G. E. Wassarman P. M. (1979). Program of early development in the mammal: Changes in the patterns and absolute rates of tubulin and total protein synthesis during oocyte growth in the mouse. Dev. Biol. 73, 120–133. 10.1016/0012-1606(79)90142-8 527765

[B134] SchwArtz A. S. K. BurKArd S. (2020). Short-term application of ibuprofen before ovulation. PMC7580263 33123693

[B135] Singh D. Irani D. Bhagat S. Vanage G. (2020a). Cypermethrin exposure during perinatal period affects fetal development and impairs reproductive functions of F1 female rats. Sci. Total Environ. 707, 135945. 10.1016/j.scitotenv.2019.135945 31863984

[B136] Singh L. Anand M. Singh S. Taneja A. (2020b). Environmental toxic metals in placenta and their effects on preterm delivery-current opinion. Drug Chem. Toxicol. 43, 531–538. 10.1080/01480545.2018.1515216 30257569

[B137] Slaby S. Hanotel J. Marchand G. Lescuyer A. Bodart J.-F. Leprêtre A. (2017). Maturation of *Xenopus laevis* oocytes under cadmium and lead exposures: Cell biology investigations. Aquat. Toxicol. 193, 105–110. 10.1016/j.aquatox.2017.10.009 29053961

[B138] Sobinoff A. P. Beckett E. L. Jarnicki A. G. Sutherland J. M. McCluskey A. Hansbro P. M. (2013). Scrambled and fried: Cigarette smoke exposure causes antral follicle destruction and oocyte dysfunction through oxidative stress. Toxicol. Appl. Pharmacol. 271, 156–167. 10.1016/j.taap.2013.05.009 23693141

[B139] Spaziani M. Tarantino C. Tahani N. Gianfrilli D. Sbardella E. Lenzi A. (2021). Hypothalamo-Pituitary axis and puberty. Mol. Cell. Endocrinol. 520, 111094. 10.1016/j.mce.2020.111094 33271219

[B140] Spinaci M. Nerozzi C. Tamanini C. Galeati G. (2020). Glyphosate and its formulation Roundup impair pig oocyte maturation. Sci. Rep. 10, 12007. 10.1038/s41598-020-68813-6 32686734 PMC7371730

[B141] Stanley J. A. Arosh J. A. Burghardt R. C. Banu S. K. (2015). A fetal whole ovarian culture model for the evaluation of CrVI-induced developmental toxicity during germ cell nest breakdown. Toxicol. Appl. Pharmacol. 289, 58–69. 10.1016/j.taap.2015.09.002 26348139 PMC4628871

[B142] Stavridis K. Triantafyllidou O. Pisimisi M. Vlahos N. (2022). Bisphenol-A and female fertility: An update of existing epidemiological studies. J. Clin. Med. 11, 7227. 10.3390/jcm11237227 36498800 PMC9736436

[B143] Stephens V. R. Rumph J. T. Ameli S. Bruner-Tran K. L. Osteen K. G. (2022). The potential relationship between environmental endocrine disruptor exposure and the development of endometriosis and adenomyosis. Front. Physiology 12, 807685. 10.3389/fphys.2021.807685 PMC8832054 35153815

[B144] Sui L. Nie J. Xiao P. Yan K. Zhang H. Liu J. (2020). Maternal benzo[a]pyrene exposure is correlated with the meiotic arrest and quality deterioration of offspring oocytes in mice. Reprod. Toxicol. 93, 10–18. 10.1016/j.reprotox.2019.12.003 31874190

[B145] Sun Y. Wang W. Guo Y. Zheng B. Li H. Chen J. (2019). High copper levels in follicular fluid affect follicle development in polycystic ovary syndrome patients: Population-based and *in vitro* studies. Toxicol. Appl. Pharmacol. 365, 101–111. 10.1016/j.taap.2019.01.008 30641075

[B146] Talbot P. Lin S. (2011). The effect of cigarette smoke on fertilization and pre-implantation development: Assessment using animal models, clinical data, and stem cells. Biol. Res. 44, 189–194. 10.4067/S0716-97602011000200011 22513422

[B147] Tao J. Yang Q. Jing M. Sun X. Tian L. Huang X. (2023). Embryonic benzophenone-3 exposure inhibited fertility in later-life female zebrafish and altered developmental morphology in offspring embryos. Environ. Sci. Pollut. Res. 30, 49226–49236. 10.1007/s11356-023-25843-7 36773251

[B148] Tchounwou P. (2004). Environmental research and public health. IJERPH 1, 1–2. 10.3390/ijerph2004010001

[B149] Thompson J. Bannigan J. (2008). Cadmium: Toxic effects on the reproductive system and the embryo. Reprod. Toxicol. 25, 304–315. 10.1016/j.reprotox.2008.02.001 18367374

[B150] Tu Z. Mu X. Chen X. Geng Y. Zhang Y. Li Q. (2019). Dibutyl phthalate exposure disrupts the progression of meiotic prophase I by interfering with homologous recombination in fetal mouse oocytes. Environ. Pollut. 252, 388–398. 10.1016/j.envpol.2019.05.107 31158667

[B151] Vimal D. Saini S. Kristipati R. R. Chowdhuri D. K. (2019). Atrazine or bisphenol A mediated negative modulation of mismatch repair gene, mlh1 leads to defective oogenesis and reduced female fertility in *Drosophila melanogaster* . Chemosphere 225, 247–258. 10.1016/j.chemosphere.2019.02.134 30877919

[B152] Vollset S. E. Goren E. Yuan C.-W. Cao J. Smith A. E. Hsiao T. (2020). Fertility, mortality, migration, and population scenarios for 195 countries and territories from 2017 to 2100: A forecasting analysis for the global burden of disease study. Lancet 396, 1285–1306. 10.1016/S0140-6736(20)30677-2 32679112 PMC7561721

[B153] Wang T. Han J. Duan X. Xiong B. Cui X.-S. Kim N.-H. (2016). The toxic effects and possible mechanisms of Bisphenol A on oocyte maturation of porcine *in vitro* . Oncotarget 7, 32554–32565. 10.18632/oncotarget.8689 27086915 PMC5078033

[B154] Wang X. Zhao X. Chen Y. Wang Q. Yang H. Xia F. (2022a). Para ‐phenylenediamine deteriorates oocyte quality by impairing mitochondrial function. Environ. Toxicol. 37, 1803–1813. 10.1002/tox.23528 35363429

[B155] Wang Y.-F. Sun X.-F. Han Z.-L. Li L. Ge W. Zhao Y. (2018). Protective effects of melatonin against nicotine-induced disorder of mouse early folliculogenesis. Aging 10, 463–480. 10.18632/aging.101405 29615536 PMC5892698

[B156] Wang Y. Wang X. Wang Y. Fan R. Qiu C. Zhong S. (2015). Effect of cadmium on cellular ultrastructure in mouse ovary. Ultrastruct. Pathol. 39, 324–328. 10.3109/01913123.2015.1027436 26107819

[B157] Wang Y. Xing C.-H. Chen S. Sun S.-C. (2022b). Zearalenone exposure impairs organelle function during porcine oocyte meiotic maturation. Theriogenology 177, 22–28. 10.1016/j.theriogenology.2021.10.008 34656833

[B158] Wang Y. Xing C.-H. Zhang H.-L. Pan Z.-N. Sun S.-C. (2021a). Exposure to nivalenol declines mouse oocyte quality via inducing oxidative stress-related apoptosis and DNA damage. Biol. Reproduction 105, 1474–1483. 10.1093/biolre/ioab171 34505141

[B159] Wang Y. Xu Y. Ju J.-Q. Liu J.-C. Sun S.-C. (2021b). Fumonisin B1 exposure deteriorates oocyte quality by inducing organelle dysfunction and DNA damage in mice. Ecotoxicol. Environ. Saf. 223, 112598. 10.1016/j.ecoenv.2021.112598 34388657

[B160] Wei K.-N. Wang X.-J. Zeng Z.-C. Gu R.-T. Deng S.-Z. Jiang J. (2021). Perfluorooctane sulfonate affects mouse oocyte maturation *in vitro* by promoting oxidative stress and apoptosis induced bymitochondrial dysfunction. Ecotoxicol. Environ. Saf. 225, 112807. 10.1016/j.ecoenv.2021.112807 34562787

[B161] Woodruff T. J. Zota A. R. Schwartz J. M. (2011). Environmental chemicals in pregnant women in the United States: Nhanes 2003-2004. Environ. Health Perspect. 119, 878–885. 10.1289/ehp.1002727 21233055 PMC3114826

[B162] World Health Organization (2023). Infertility prevalence estimates, 1990–2021. Available at: https://www.who.int/publications-detail-redirect/978920068315 (Accessed April 21, 2023).

[B163] Wright D. L. Afeiche M. C. Ehrlich S. Smith K. Williams P. L. Chavarro J. E. (2015). Hair mercury concentrations and *in vitro* fertilization (IVF) outcomes among women from a fertility clinic. Reprod. Toxicol. 51, 125–132. 10.1016/j.reprotox.2015.01.003 25601638 PMC4425999

[B164] Wu S.-C. Liu M. (2012). *In vitro* assessment of reproductive toxicity of cigarette smoke and deleterious consequences of maternal exposure to its constituents. Biol. Res. 45, 101–109. 10.4067/S0716-97602012000200001 23096353

[B165] Xiao Y. Yuan B. Hu W. Qi J. Jiang H. Sun B. (2021). Tributyltin oxide exposure during *in vitro* maturation disrupts oocyte maturation and subsequent embryonic developmental competence in pigs. Front. Cell. Dev. Biol. 9, 683448. 10.3389/fcell.2021.683448 34262900 PMC8273238

[B166] Yahfoufi Z. A. Bai D. Khan S. N. Chatzicharalampous C. Kohan-Ghadr H.-R. Morris R. T. (2020). Glyphosate induces metaphase II oocyte deterioration and embryo damage by zinc depletion and overproduction of reactive oxygen species. Toxicology 439, 152466. 10.1016/j.tox.2020.152466 32315717

[B167] Yang L. Baumann C. De La Fuente R. Viveiros M. M. (2020). Mechanisms underlying disruption of oocyte spindle stability by bisphenol compounds. Reproduction 159, 383–396. 10.1530/REP-19-0494 31990668 PMC7032969

[B168] Yang S. Wang Y. Zhang L. Ding Z. Zhou X. Duan Z. (2023). High-dose synthetic phenolic antioxidant propyl gallate impairs mouse oocyte meiotic maturation through inducing mitochondrial dysfunction and DNA damage. Environ. Toxicol. 38, 1800–1810. 10.1002/tox.23807 37052413

[B169] Yin S. Tang M. Chen F. Li T. Liu W. (2017). Environmental exposure to polycyclic aromatic hydrocarbons (PAHs): The correlation with and impact on reproductive hormones in umbilical cord serum. Environ. Pollut. 220, 1429–1437. 10.1016/j.envpol.2016.10.090 27838061

[B170] Zhai Q.-Y. Ge W. Wang J.-J. Sun X.-F. Ma J.-M. Liu J.-C. (2018). Exposure to Zinc oxide nanoparticles during pregnancy induces oocyte DNA damage and affects ovarian reserve of mouse offspring. Aging 10, 2170–2189. 10.18632/aging.101539 30153657 PMC6128443

[B171] Zhan C. Cao X. Zhang T. Guo J. Xu G. Wang H. (2022). Melatonin protects porcine oocyte from copper exposure potentially by reducing oxidative stress potentially through the Nrf2 pathway. Theriogenology 193, 1–10. 10.1016/j.theriogenology.2022.09.004 36115287

[B172] Zhang J. Zhao C. Shi F. Zhang S. Wang S. Feng X. (2021). Melatonin alleviates the deterioration of oocytes and hormonal disorders from mice subjected to glyphosate. Mol. Cell. Endocrinol. 520, 111073. 10.1016/j.mce.2020.111073 33159990

[B173] Zhang M. Miao Y. Chen Q. Cai M. Dong W. Dai X. (2018). BaP exposure causes oocyte meiotic arrest and fertilization failure to weaken female fertility. FASEB J. 32, 342–352. 10.1096/fj.201700514r 28904021

[B174] Zhang S.-X. Ding Z.-M. Ahmad M. J. Wang Y.-S. Duan Z.-Q. Miao Y.-L. (2020). Bisphenol B exposure disrupts mouse oocyte meiotic maturation *in vitro* through affecting spindle assembly and chromosome alignment. Front. Cell. Dev. Biol. 8, 616771. 10.3389/fcell.2020.616771 33392205 PMC7773771

[B175] Zhang W. Liu Y. An Z. Huang D. Qi Y. Zhang Y. (2011). Mediating effect of ROS on mtDNA damage and low ATP content induced by arsenic trioxide in mouse oocytes. Toxicol. Vitro 25, 979–984. 10.1016/j.tiv.2011.03.009 21419842

[B176] Zhang X.-F. Zhang L.-J. Li L. Feng Y.-N. Chen B. Ma J.-M. (2013). Diethylhexyl phthalate exposure impairs follicular development and affects oocyte maturation in the mouse: Diethylhexyl Phthalate. Environ. Mol. Mutagen. 54, 354–361. 10.1002/em.21776 23625783

[B177] Zhiqiang E. Zhao Y. Sun J. Zhang X. Jin Q. Gao Q. (2022). Glyphosate decreases bovine oocyte quality by inducing oxidative stress and apoptosis. Zygote 30, 704–711. 10.1017/S0967199422000181 35677960

[B178] Zhou C. Flaws J. A. (2016). Effects of an environmentally relevant phthalate mixture on cultured mouse antral follicles. Toxicol. Sci. 156, 217–229. kfw245. 10.1093/toxsci/kfw245 PMC6075604 28013214

[B179] Zhou W. Niu Y.-J. Nie Z.-W. Kim Y.-H. Shin K.-T. Guo J. (2019). Fipronil induces apoptosis and cell cycle arrest in porcine oocytes during *in vitro* maturation. Apoptosis 24, 718–729. 10.1007/s10495-019-01552-w 31240517

[B180] Zhu J.-Q. Liu Y. Zhang J.-H. Liu Y.-F. Cao J.-Q. Huang Z.-T. (2018). Cadmium exposure of female mice impairs the meiotic maturation of oocytes and subsequent embryonic development. Toxicol. Sci. 164, 289–299. 10.1093/toxsci/kfy089 29684212

