# Supplementary material for: Fertility loss: negative effects of environmental toxicants on oogenesis
Source: Front Physiol. 2023 Aug 4;14:1219045. doi: 10.3389/fphys.2023.1219045 (PMC10436557; doi:10.3389/fphys.2023.1219045)
Supplement: Supplementary file 1 [file Table1.docx]

**Supplementary Table 1. Toxic effects of various environmental toxicants on oocytes**

| Categories of environmental toxicants | | | Toxic effects on oocytes | Mechanisms | Experimental subjects | References |
| --- | --- | --- | --- | --- | --- | --- |
| Heavy metal |  | Cd | Inhibition of oocyte meiosis,  Reduction in the quantity and quality of oocytes,  Formation of aneuploid oocytes. | Abnormal cytoskeletal organization, interference with regular spindle assembly, chromosome arrangement and actin cap development,  Abnormal mitochondrial distribution and function, with decreased ATP content,  Disruption of histone modifications,  Increased levels of ROS,  Apoptosis and DNA damage. | Mouse | (Liu et al., 2018a; Zhu et al., 2018; Cheng et al., 2019; Dong et al., 2020) |
|  |  | As | Inhibition of oocyte meiosis,  Reduction in the quantity and quality of oocytes,  Inhibition of the quality of embryonic development. | Reduction in the copy number of mtDNA,  Abnormal mitochondrial distribution and function, with decreased ATP content,  Increased levels of ROS,  Autophagy,  Abnormal expression of 5mC, H3K4me3 and H3K9me3. | Mouse,  Goat,  Pig | (Zhang et al., 2011; Ommati et al., 2020; Nava-Rivera et al., 2021; Kang et al., 2022) |
|  |  | Pb | Inhibition of oocyte maturation and fertilization. | Induction of oxidative stress,  Activation of the Nrf2/Keap1 pathway,  Decreased activity of catalase, glutathione peroxidase, total superoxide dismutase and glutathione-S-transferase in the ovaries and increased malondialdehyde levels. | Mouse,  Buffalo,  Xenopus laevis | (Nandi et al., 2010; Slaby et al., 2017; Jiang et al., 2021a) |
|  |  | Hg | Reduction in the quantity of oocytes. | Atresia of the previtellogenic oocytes,  Reduction in the total number of primordial, primary and Graaf follicles,  A significant increase in the total volume of atretic follicles. | Zebrafish,  Rat | (Altunkaynak et al., 2016; Patel et al., 2022) |
|  |  | Cu | Reduction in the quality of oocytes. | Abnormal chromosome structure of oocytes and abnormal dynamics of actin distribution,  Activation of the Nrf2 signaling pathway,  Induction of oxidative stress,  Increased levels of ROS. | Pig,  Sheep | (Chen et al., 2021; Zhan et al., 2022; Ren et al., 2023) |
|  |  | Other metal | Reduction in the quantity and quality of oocytes,  Inhibition of the quality of embryonic development. | Increased levels of ROS,  Abnormal mitochondrial distribution and function, with decreased ATP content,  Disruption of cellular iron homeostasis,  Alteration of cell cycle regulatory genes and proteins,  Apoptosis. | Mouse | (Stanley et al., 2015; Xiao et al., 2021) |
| Cigarette smoke |  | Smoke | Delayed cleavage of the ovum,  Impairing early embryonic development,  Impairing the development of oocytes,  Reduction in follicular survival, | Shorter and wider spindle bodies,  Induction of oxidative stress,  Changes in mid-term II spindle bodies,  Changes in miRNAs,  Activated *CYP2E1*,  Increased risk of DNA damage and abnormal spindle morphology,  Decreased levels of 5mC and H3K4me2,  Apoptosis,  Abnormal mitochondrial distribution and function,  Increased levels of ROS,  Premature luteinization of follicles,  Decreased diameter of oocytes without zona pellucida,  Abnormal PB1. | Human,  Mouse | (Jennings et al., 2011; Sadeu and Foster, 2011a; Paixão et al., 2012; Fréour et al., 2013; Sobinoff et al., 2013; Mai et al., 2014; Camlin et al., 2016; Budani et al., 2017, 2019; Liu et al., 2019b, 2019a) |
|  |  | Nicotine | Reduction in the quantity and quality of oocytes. | Induction of oxidative stress,  Autophagy,  Apoptosis,  Upregulation of AMPKα -1, increased LC 3-II/LC 3-I ratio, and downregulation of AKT and mTOR,  Reduced expression of oocyte-specific genes such as *Nobox, Lhx 8, Figla* and *Sohlh 2.* | Human,  Mouse | (Cheng et al., 2018, 2022; Wang et al., 2018; Liu et al., 2020) |
|  |  | PAH | Inhibition of oocyte meiosis,  Reduction in the quantity and quality of oocytes,  Formation of aneuploid oocytes,  Inhibition of oocyte maturation,  Reduced fertilization rate. | Increased follicular atresia,  Apoptosis,  Upregulation of the PI3K/Akt signalling pathway,  Disruption of the calcium signalling pathway,  Abnormal mitochondrial function and decreased ATP content in offspring oocytes,  Accumulation of ROS in offspring oocytes,  Increased genomic 5 mC levels in offspring oocytes,  Disruption of normal spindle assembly, chromosome arrangement and mitotic-microtubule attachment,  Reduction in the number of sperm bound to the zona pellucida and interference with the gamete fusion process. | Chicken,  Mouse | (Sadeu and Foster, 2011b; Ge et al., 2012; Einaudi et al., 2014; Zhang et al., 2018, 2018; Sui et al., 2020; Guo et al., 2022; Malott et al., 2022) |
| Agricultural and industrial toxicants | Herbicides | Glyphosate-based herbicides | Reduction in the quantity and quality of oocytes,  Inhibition of oocyte maturation,  Influence on early embryonic oogenesis. | Disruption of microtubule tissue centres and chromosomes,  Depletion of intracellular zinc bioavailability,  Increased levels of ROS,  Spindle deficiency and chromosomal misalignment. | Mouse,  Pig,  Cattle | (Spinaci et al., 2020; Yahfoufi et al., 2020; Cao et al., 2021; Zhang et al., 2021; E et al., 2022) |
|  |  | Fenoxaprop-ethyl | Inhibition of oocyte meiosis. | Abnormal cytoskeletal organization,  Increased levels of ROS. | Mouse | (He et al., 2019) |
|  |  | Atrazine | Reduction in the quantity and quality of oocytes,  Inhibition of the quality of embryonic development,  Inhibition of oocyte maturation. | Reduction in the number of primordial follicles,  Increased incidence of polyzygotic follicles in adult mice,  Downregulation of MLH1,  Transcriptional and translational deficiency,  Reduction of double-strand breaks and association complex formation,  Apoptosis. | Mouse,  Drosophila melanogaster | (Gely-Pernot et al., 2017; Vimal et al., 2019) |
|  | Pesticides | organophosphorus pesticides | Reduction in the quantity and quality of oocytes,  Inhibition of oocyte maturation,  Inhibition of oocyte meiosis,  Reduced fertilization rate. | Reduction in the number of primordial follicles,  Affecting the regulation of genes for transcription, translation-related proteins and mitochondrial function,  Spindle malformation,  Depletion of glutathione,  Reduction in the extrusion rate of PB1 and damaged MII oocytes,  Apoptosis. | Mouse,  Pig | (Bonilla et al., 2008; Nair et al., 2014; Flores et al., 2017; Han et al., 2018; Jiang et al., 2021b; Gai et al., 2022; Satar et al., n.d.) |
|  |  | Neonicotinoids | Reduction in the quantity and quality of oocytes,  Inhibition of oocyte maturation. | Chromosomal abnormalities,  Induction of oxidative stress,  Affecting the expression of several oocyte genes associated with inflammation, apoptosis and endoplasmic reticulum stress,  Affecting the integrity of DNA,  Inducing endoplasmic reticulum stress. | Mouse,  Pig | (Ishikawa et al., 2015; Liu et al., 2021) |
|  |  | Formate insecticides | Reduction in the quantity and quality of oocytes. | Inhibition of polar body extrusion in oocytes,  Increased superoxide anion radicals in oocytes and a significant decrease in mitochondrial membrane potential in mid-stage II oocytes,  Abnormal morphology of spindle bodies,  Impaired periplasmic protein orientation. | Mouse | (Cinar et al., 2015; He et al., 2022a) |
|  |  | Pyrethroids | Inhibition of oocyte maturation. | Apoptosis,  Induction of oxidative stress,  DNA double-strand breaks. | Mouse,  Pig | (Petr et al., 2013; Jia et al., 2019b) |
|  |  | FPN | Reduction in the quantity and quality of oocytes. | DNA damage,  Apoptosis,  Increased levels of ROS,  Cell cycle stasis. | Pig | (Zhou et al., 2019) |
|  |  | MXC | Inhibition of oocyte maturation. | Increased levels of ROS. | Mouse | (Liu et al., 2016) |
|  |  | Rotenone | Inhibition of oocyte maturation. | Mitochondrial dysfunction. | Pig | (Heo et al., 2022) |
| s | Fungicide |  | Inhibition of oocyte maturation,  Inhibition of oocyte meiosis,  Reduction in the quantity and quality of oocytes,  Reduced fertilization rate,  Impairing early embryonic development,  Reduction in the number of oocytes in the F1 generation. | Abnormal cytoskeletal organization, interference with regular spindle assembly, chromosome arrangement,  Abnormal mitochondrial distribution and function, with decreased ATP content,  Induction of oxidative stress,  Increased levels of ROS,  Autophagy,  Apoptosis and DNA damage. | Mouse,  Goat | (Esmaiel et al., 2019; Gao et al., 2022; He et al., 2022b) |
|  | Plasticizers | DEHP | Inhibition of oocyte meiosis,  Reduction in the quantity of oocytes. | Affecting primordial follicle production,  Decreasing and/or delaying the methylation level of imprinted genes in oocytes,  Abnormal mid-phase II spindle in mature oocytes in vitro,  Alteration of miRNA expression in F1 generation ovaries,  Reduction in the expression of H3K4me3, ER VII., PR and Notch2 signalling components. | Mouse | (Zhang et al., 2013; Mu et al., 2015; Zhou and Flaws, 2016; Mirihagalle et al., 2019; Gonsioroski et al., 2022; Li et al., 2023) |
|  |  | DBP | Inhibition of oocyte meiosis,  Reduction in the quantity of oocytes. | Reducing germinal vesicle breakdown and polar body extrusion,  Disruption of cytoskeletal dynamics and inhibition of spindle stability,  Increased levels of ROS,  Apoptosis,  DNA damage. | Mouse | (Tu et al., 2019; Li et al., 2019) |
|  |  | BPA | Inhibition of oocyte meiosis,  Reduction in the quantity of oocytes,  Impairing embryonic development,  Inhibition of oocyte maturation. | Activation of the Gper/Egfr/Mapk 3/1 pathway,  Disruption of cytoskeletal dynamics and inhibition of spindle stability,  Affecting epigenetic modifications,  Apoptosis and autophagy,  Affecting organelles in oocytes, including mitochondrial dysfunction and abnormal mitochondrial distribution, abnormal ER distribution, abnormal Golgi organelle structure and lysosomal damage,  Affecting AMH and AMH receptor expression. | Zebrafish,  Mouse,  Cattle,  Pig | (Ferris et al., 2016; Nakano et al., 2016; Wang et al., 2016; Yang et al., 2020; Pan et al., 2021; Saleh et al., 2021; Fitzgerald et al., n.d.) |
|  |  | BPA alternatives | Reduction in the quantity of oocytes,  Inhibition of oocyte maturation. | Abnormal spindle assembly,  Decreased ATP level,  Increased levels of ROS,  Apoptosis,  Affecting AMH and AMH receptor expression,  Disturbance of CG distribution in oocytes. | Pig,  Mouse,  Cattle | (Ding et al., 2017; Jia et al., 2019a; Jiao et al., 2020; Prokešová et al., 2020; Saleh et al., 2021) |
| PPCPs | NP | ZnO | Inhibition of oocyte meiosis,  Reduction in the quantity and quality of oocytes,  Impairing embryonic development. | Abnormal mitochondrial distribution and function, with decreased ATP content,  Abnormal cytoskeletal organization, interference with regular spindle assembly,  Reduction in the extrusion rate of PB1,  Increased levels of ROS,  Autophagy,  Apoptosis,  Affecting cell-oocyte-complex expansion. | Mouse | (Zhai et al., 2018; Camaioni et al., 2021; Huang et al., 2022) |
|  | PBs | MeBP | Inhibition of oocyte maturation. | Affecting cell-oocyte-complex expansion,  Altering oocyte morphology. | Pig | (Barajas‐Salinas et al., 2021) |
|  |  | IBP | Inhibition of oocyte meiosis,  Reduction in the quantity and quality of oocytes. | Abnormal cytoskeletal organization, interference with regular spindle assembly,  Affecting cell-oocyte-complex expansion,  Increased levels of ROS,  Apoptosis,  elevated levels of H3K9me3 and H3K27me3. | Pig | (Meng et al., 2020) |
|  |  | BP | Inhibition of oocyte meiosis,  Inhibition of oocyte maturation. | Affecting cell-oocyte-complex expansion,  Abnormal mitochondrial distribution and function, with decreased ATP content,  Increased levels of ROS,  Autophagy,  Apoptosis,  higher expression of γ-H2AX, annexin V positivity and LC3. | Pig | (Jeong et al., 2020) |
|  | Others | PPD | Inhibition of oocyte meiosis,  Inhibition of oocyte maturation. | Abnormal mitochondrial distribution and function, with decreased ATP content,  Abnormal cytoskeletal organization, interference with regular spindle assembly,  Increased levels of ROS,  Apoptosis,  Absence of Juno proteins. |  | (Wang et al., 2022) |
|  |  | PG | Inhibition of oocyte meiosis,  Inhibition of oocyte maturation. | Increased levels of ROS,  Apoptosis,  Elevated levels of H3K27me2 and H3K27me3. |  | (Yang et al., 2023) |
|  | Organic ultraviolet filters | OBZ | Inhibition of oocyte meiosis,  Reduction in the quantity of oocytes,  Inhibition of oocyte maturation. | Increasing the expression of the Kdm5 family of genes,  Abnormal cytoskeletal organization, interference with regular spindle assembly, chromosome arrangement,  Abnormal mitochondrial distribution and function, with decreased ATP content,  Increased levels of ROS,  Apoptosis. | Mouse | (Jin et al., 2021) |
|  |  | BP3 | Inhibition of oocyte maturation. | Abnormal cytoskeletal organization, interference with regular spindle assembly, chromosome arrangement,  Abnormal mitochondrial distribution and function, with decreased ATP content,  Increased levels of ROS. | Zebrafish | (Tao et al., 2023) |
|  |  | OCL | Inhibition of oocyte meiosis,  Reduction in the quantity of oocytes,  Inhibition of oocyte maturation. | Abnormal cytoskeletal organization, interference with regular spindle assembly, chromosome arrangement,  Abnormal mitochondrial distribution and function, with decreased ATP content,  Increased levels of ROS,  Apoptosis. | Mouse | (Chang et al., 2022) |
|  |  | EE2 | Inhibition of oocyte maturation. | Reduction of Juno proteins,  Increased levels of ROS,  Apoptosis. | Mouse | (Dai et al., 2020) |
| Food toxicants | Mycotoxins | Fusarium mycotoxin | Inhibition of oocyte meiosis,  Inhibition of oocyte maturation. | Abnormal cytoskeletal organization, interference with regular spindle assembly, chromosome arrangement,  Abnormal mitochondrial distribution and function, with decreased ATP content,  Increased levels of ROS,  Apoptosis,  Autophagy,  Reduction in the extrusion rate of PB1,  Reduced p-MAPK protein levels,  Abnormal Lamp2, LC3 and mTOR mRNA expression,  Elevated levels of H3K27me3 and H3K4me2 proteins and related methyltransferase gene mRNA levels,  altered ER stress levels and reduced expression of GRP78. | Pig,  Mouse | (Han et al., 2016; Schoevers et al., 2016; Wang et al., 2021a, 2021b) |
|  |  | OTA | Inhibition of oocyte meiosis,  Reduction in the quality and quantity of oocytes,  Inhibition of oocyte maturation. | Abnormal cytoskeletal organization, interference with regular spindle assembly, chromosome arrangement,  Abnormal mitochondrial distribution and function, with decreased ATP content,  Increased levels of ROS,  Autophagy,  Apoptosis,  Altered levels of 5mc, 5hmC, H3K9ac and H3K9me3. | Mouse | (Huang and Chan, 2016; Jia et al., 2020; Lan et al., 2020) |
|  | Other food toxicants | 4-MI | Inhibition of oocyte meiosis,  Reduction in the quantity of oocytes,  Inhibition of oocyte maturation. | Abnormal cytoskeletal organization, interference with regular spindle assembly, chromosome arrangement,  Abnormal mitochondrial distribution and function, with decreased ATP content,  Increased levels of ROS,  Apoptosis. | Mouse | (Lu et al., 2022) |
|  |  | α-solanine | Inhibition of oocyte meiosis,  Reduction in the quantity of oocytes. | Autophagy,  Apoptosis,  Increased level of autophagy-related genes (*LC3, ATG7,* and *LAMP2*) and apoptosis-related genes (*BAX* and *CASP3*),  Increased the levels of H3K36me3 and H3K27me3. | Pig | (Lin et al., 2018) |
|  |  | Arecoline | Inhibition of oocyte meiosis,  Reduction in the quantity of oocytes. | Abnormal cytoskeletal organization, interference with regular spindle assembly, chromosome arrangement,  Abnormal mitochondrial distribution and function, with decreased ATP content,  Increased levels of ROS,  Apoptosis. | Mouse | (Li et al., 2020) |
| Fluoride |  | Fluoride salt | Inhibition of oocyte meiosis,  Reduction in the quantity of oocytes,  Inhibition of oocyte maturation. | Abnormal cytoskeletal organization, interference with regular spindle assembly, chromosome arrangement,  Abnormal mitochondrial distribution and function, with decreased ATP content,  Increased levels of ROS,  Apoptosis,  Decreased levels of H3K9ac and H3K18ac,  Reduced the expression of oocyte-specific genes involved in oocyte growth and induction of the acrosome response. | Pig,  Mouse | (Yin et al., 2015; Liang et al., 2016, 2017; Liu et al., 2018b) |
|  |  | Perfluorinated compound | Inhibition of oocyte meiosis,  Reduction in the quantity of oocytes. | Abnormal cytoskeletal organization, interference with regular spindle assembly, chromosome arrangement,  Abnormal mitochondrial distribution and function, with decreased ATP content,  Increased levels of ROS,  Apoptosis,  Reduction in the extrusion rate of PB1,  Disrupting oocyte calcium homeostasis and gap-junctional intercellular communication. | Pig,  Mouse | (Domínguez et al., 2019; Martínez-Quezada et al., 2021; Wei et al., 2021) |
